# *Malassezia restricta*‐Derived Extracellular Vesicles Drive Ovarian Cancer Progression Through JAK2/STAT3‐Mediated M2 Macrophage Polarisation

**DOI:** 10.1111/1751-7915.70396

**Published:** 2026-06-05

**Authors:** Ying Jiang, Fen Wei, Qingling Yang, Xue Wu, Qifa Huang, Ang Dai, Qi Chen, Tingtao Chen

**Affiliations:** ^1^ Department of Obstetrics and Gynecology, The Second Affiliated Hospital, Jiangxi Medical College Nanchang University Nanchang Jiangxi China; ^2^ Jiangxi Key Laboratory of Molecular Medicine, The Second Affiliated Hospital of Nanchang University Nanchang University Nanchang China; ^3^ Jiangxi Province Key Laboratory of Bioengineering Drugs, School of Pharmacy, Jiangxi Medical College Nanchang University Nanchang China; ^4^ National Engineering Research Center for Bioengineering Drugs and the Technologies, Institute of Translational Medicine, Jiangxi Medical College Nanchang University Nanchang China

**Keywords:** epithelial ovarian cancer, extracellular vesicles, JAK2/STAT3 signalling pathway, macrophage polarisation, *Malassezia restricta*

## Abstract

The intratumoural mycobiome is a critical constituent of the tumour microenvironment; however, its specific impact on epithelial ovarian cancer (EOC) progression and the underlying molecular mechanisms remain largely elusive. In this study, internal transcribed spacer 1 (ITS1) sequencing revealed a significant enrichment of *Malassezia* in EOC tissues compared with epithelial borderline ovarian tumours, with its abundance positively correlated with disease progression. Subsequent intratumoural microbiota transplantation and mono‐colonisation in a murine EOC model demonstrated that *Malassezia restricta* substantially accelerated tumour growth and increased M2 macrophage infiltration. Furthermore, in vitro assays established that *M. restricta*‐derived extracellular vesicles (MrEVs) play a pivotal role in inducing M2 macrophage polarisation. Mechanistically, both in vitro and in vivo data showed that MrEVs activate the Janus kinase 2/signal transducer and activator of transcription 3 (JAK2/STAT3) signalling pathway, thereby driving M2 polarisation and tumour malignancy. Collectively, these findings identify *M. restricta* as a pro‐tumourigenic fungus in EOC and uncover a previously unrecognised fungal–immune axis that promotes tumour progression. This study provides new insight into the oncogenic role of tumour‐resident fungi and highlights the *M. restricta* EV–JAK2/STAT3 axis as a potential therapeutic target for immune modulation.

## Graphical abstract

Schematic diagram of the potential mechanism by which *Malassezia restricta* promote ovarian cancer progression. *Malassezia restricta*‐derived extracellular vesicles promoted epithelial ovarian cancer progression by inducing M2 macrophage polarisation via activation of the JAK2/STAT3 signalling pathway.

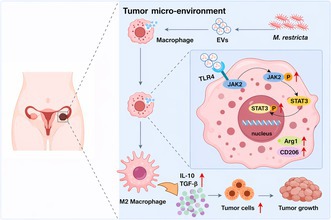

## 1 Introduction

Epithelial ovarian cancer (EOC), which accounts for the majority of ovarian cancer cases, is characterised by aggressive behaviour, a silent onset and poor clinical outcomes (Hollis 2023). In 2022, approximately 324,603 new cases (1.7%) and 206,956 deaths (2.1%) from EOC were reported worldwide, making it the leading cause of mortality among gynaecological cancers (Bray et al. 2024; Webb and Jordan 2024). Despite extensive research into genetic alterations, epigenetic dysregulation and tumour microenvironment (TME) remodelling in EOC (Savant et al. 2018; Lapke et al. 2021; Wang, Wang, et al. 2025; Wang, Zhu, et al. 2025), the fundamental factors driving disease progression remain incompletely understood. A deeper understanding of the underlying molecular mechanisms is therefore essential for identifying new therapeutic strategies and improving patient outcomes.

The tumour‐resident microbiome has recently attracted considerable attention because of its critical role in oncogenesis (Yang et al. 2023; Zhang, Feng, et al. 2025; Zhang, Huang, et al. 2025; Zhang, Xiang, et al. 2025; Zhang, You, et al. 2025). Tumour tissues were historically regarded as sterile; however, advances in next‐generation sequencing and imaging technologies have revealed substantial microbial colonisation across multiple malignancies (Cao et al. 2024), including pancreatic (Abe et al. 2024), breast (Fu et al. 2022), lung (Ochi et al. 2025) and ovarian cancers (Qin et al. 2024). Nejman et al. (2020) demonstrated that ovarian cancer harbours a distinct intratumoural microbial profile characterised by a significant enrichment of tumour‐specific bacteria compared with normal ovarian tissue. Our previous work further showed that *Propionibacterium acnes* is a key intratumoural bacterium that promotes EOC progression through activation of the Hedgehog (Hh) signalling pathway (Huang et al. 2022). Beyond bacteria, fungi are increasingly recognised as important components of the intratumoural microbiome. Growing evidence indicates that they influence tumour biology by shaping immune responses, sustaining chronic inflammation, and altering the TME (Bahuguna and Dubey 2023; Zhang, Feng, et al. 2025; Zhang, Huang, et al. 2025; Zhang, Xiang, et al. 2025; Zhang, You, et al. 2025). Notably, Dohlman et al. (2022) identified fungal communities within tumours across 35 cancer types. In addition, Alam et al. (2022) reported that tumour‐associated fungi induce interleukin (IL)‐33 release and trigger type 2 immune responses, thereby facilitating pancreatic tumour development. Similarly, recent work showed that *Aspergillus sydowii* promotes lung adenocarcinoma progression by establishing an immunosuppressive microenvironment via the Dectin‐1/CARD9 pathway (Liu, Li, et al. 2023; Liu, Yi, et al. 2023). However, whether intratumoural fungi are present in EOC and how they contribute to disease progression remain unclear.

Extracellular vesicles (EVs) are nanoscale, membrane‐bound particles released by a wide range of prokaryotic and eukaryotic organisms (Kalluri and LeBleu 2020). Due to their complex cargo of proteins, nucleic acids, lipids and metabolites, EVs act as key mediators of communication between cells and their surrounding environment (Kumar et al. 2024). EVs can be taken up by host cells through phagocytosis, endocytosis, membrane fusion or micropinocytosis, after which they deliver their contents to regulate immune responses, maintain epithelial barrier integrity and modulate tumour progression (Marar et al. 2021; Liu and Wang 2023). Recent studies have shown that EVs derived from *Fusobacterium nucleatum* can polarise tumour‐associated macrophages (TAMs) towards a less aggressive phenotype. This effect limits cytotoxic T‐cell infiltration and accelerates the progression of head and neck squamous cell carcinoma (Li et al. 2025). Fungal‐derived EVs likewise exhibit potent immunomodulatory properties. Brown Harding et al. (2024) demonstrated that nucleic acids within *Candida albicans* EVs induce type I interferon responses in host immune cells through activation of the cyclic GMP–AMP synthase–stimulator of interferon genes (cGAS–STING) pathway. Moreover, Xu, Ding, et al. (2025), Xu, Luo, et al. (2025) and Xu, Qiao, et al. (2025) showed that PMA1‐containing EVs from *C. albicans* drive dendritic cell activation and T helper 17 responses, thereby exacerbating colitis via a CARD9‐dependent mechanism. However, the role of fungal‐derived EVs in EOC remains poorly defined, and their direct contribution to disease progression is largely unknown.

In this study, internal transcribed spacer 1 (ITS1) sequencing was performed on tumour samples from patients with EOC and epithelial borderline ovarian tumours (EBOT), revealing a markedly higher abundance of *Malassezia* in EOC tissue. In a subcutaneous EOC mouse model, intratumoural injection of either mixed microbiota derived from patients with EOC or *Malassezia restricta* alone significantly accelerated tumour growth and increased infiltration of M2 macrophages. In vitro analyses further demonstrated that *M. restricta*‐derived EVs strongly induce M2 macrophage polarisation. Mechanistically, both in vitro and in vivo data supported a model in which EVs from *M. restricta* promote M2 macrophage polarisation and tumour progression through activation of the Janus kinase 2/signal transducer and activator of transcription 3 (JAK2/STAT3) signalling pathway. Collectively, these findings identify, for the first time, a functional role for *M. restricta* and its EVs in shaping the tumour immune microenvironment (TIME) and driving EOC progression, highlighting their potential as novel biomarkers and therapeutic targets.

## 2 Materials and Methods

### 2.1 Patients and Samples

The Ethics Committee of the Second Affiliated Hospital of Nanchang University granted ethical approval for this research (no. 2024076). Additionally, written informed consent was acquired from every participant. Between August 2024 and August 2025, 40 women of Chinese ancestry (20 with EOC and 20 with EBOT), aged 18–75 years, were enrolled.

Patients were enrolled according to the following criteria. The EOC group included patients with primary epithelial ovarian cancer diagnosed preoperatively and confirmed by final postoperative pathological evaluation. Eligible patients had not received antibiotics, probiotics or other related medications within 3 months before surgery and had no serious comorbidities. Patients were excluded if they had significant cardiac, pulmonary, hepatic or renal dysfunction; metabolic instability; a history of infectious, immunosuppressive, haematological or other malignant diseases; chronic inflammatory disorders; current pregnancy; or use of antibiotics or Traditional Chinese Medicine within the preceding 3 months. EBOT represents a distinct subtype of ovarian epithelial neoplasms characterised by increased epithelial proliferation and moderate cytological atypia beyond those of benign ovarian tumours, yet in the absence of destructive stromal invasion. The EBOT group included patients with pathologically confirmed epithelial borderline ovarian tumours on postoperative examination, including serous and mucinous subtypes. All enrolled EBOT patients were aged 18–75 years, were treatment‐naïve at initial diagnosis, and had not received any prior interventions for ovarian lesions before study entry.

### 2.2 Collection of Human Tumour Tissue Samples

Ovarian tumour tissues were excised under sterile conditions by the same surgeon and confirmed by a pathologist. To minimise contamination, all samples were handled using sterile materials throughout the collection procedure. Immediately after excision, the specimens were transferred into sterile tubes containing 30% glycerol preservation solution and stored at −80°C until subsequent analysis.

### 2.3 Cell Culture and Stimulation

The ID8 cells (RRID: CVCL_IU14) were purchased from Fuxiang Biotechnology (Cat# XF1030, Shanghai, China), and the RAW 264.7 cells (RRID: CVCL_0493) were obtained from Servicebio Technology (Cat# STCC20020, Wuhan, China). Both cell lines were maintained in high‐glucose DMEM (Gibco, USA) supplemented with 10% foetal bovine serum (Gibco, USA) and incubated at 37°C in a humidified atmosphere containing 5% CO_2_. The identity of the cell lines was authenticated by the suppliers via short tandem repeat (STR) profiling; specifically, the ID8 cells showed a 100% match with the reference genotype, and the RAW 264.7 cells exhibited a 95.20% match with their respective reference profiles. We confirm that neither cell line is listed in the ICLAC database of misidentified or contaminated cultures. Furthermore, all cells were confirmed to be free of mycoplasma contamination prior to use in the described experiments. Detailed metadata and authentication parameters for these cell lines are summarised in Table S1.

To identify the fungal components responsible for inducing M2 macrophage polarisation, RAW264.7 cells were treated with either the culture supernatant or heat‐inactivated cell bodies of *M. restricta* for 24 h. For MrEV intervention and pathway validation experiments, RAW264.7 cells were treated with MrEVs (20 μg/mL) for 24 h. In the inhibitor experiments, cells were pretreated with the JAK2 inhibitor AG490 (50 μM) for 4 h before MrEV stimulation. Detailed information on AG490 is provided in Table S2.

### 2.4 Preparation of Intratumoural Mixed Microbiota From EBOT and EOC Tissues

Tumour tissues from patients with EBOT or EOC were collected under sterile conditions and processed under low‐temperature conditions. Tissues were homogenised in batches using a tissue homogeniser on ice. Microbial‐enriched pellets were obtained by differential centrifugation. Briefly, homogenates were first centrifuged at 300 × *g* for 5 min to remove large tissue debris. Supernatants were then filtered through a 40 μm nylon mesh to eliminate residual cell clumps, followed by centrifugation at 12,000 × *g* for 15 min to collect pellets. After discarding supernatants, pellets were resuspended and washed with sterile normal saline to obtain microbial‐enriched suspensions for subsequent intratumoural injection. To minimise batch‐to‐batch variation, all suspensions were prepared at a uniform tissue weight‐to‐volume ratio under consistent conditions. Final suspensions were aliquoted and stored at −80°C until use. For each injection, a fresh aliquot was thawed and used immediately to avoid repeated freeze–thaw cycles.

### 2.5 Animal Model and Experimental Design

Female C57BL/6 mice (6–8 weeks old, 16–18 g) were purchased from Sibeifu Biotechnology (Beijing, China) and acclimatised for 1 week under specific pathogen‐free conditions. After shaving and disinfection, 3 × 10^6^ ID8 cells were inoculated subcutaneously into the right dorsal flank of each mouse. All animal experiments were approved by the Animal Care Committee of Nanchang University (no. NCULAE‐20221031067).

#### 2.5.1 Experiment 1

Effect of EBOT‐ and EOC‐derived intratumoural microbiota on tumour progression. A total of 36 mice were randomly assigned to four groups (*n* = 9 per group): (1) M group: Mice received oral gavage of PBS once daily for seven consecutive days before tumour inoculation. On Day 0, mice were inoculated subcutaneously with ID8 cells, followed by a 4‐week period to allow tumour establishment. The mice then received intratumoural injection of PBS once every 3 days for 4 weeks, while normal drinking water was maintained throughout the experiment. (2) AmB group: Mice received oral gavage of amphotericin B (AmB, 0.1 mg/mL) once daily for seven consecutive days before tumour inoculation. On Day 0, mice were inoculated subcutaneously with ID8 cells, followed by a 4‐week period to allow tumour establishment. During this 4‐week period, the mice received AmB‐supplemented drinking water (0.5 μg/mL). The mice then received intratumoural injection of PBS once every 3 days for 4 weeks, while normal drinking water was maintained throughout the treatment period. (3) BT group: Mice received oral gavage of AmB (0.1 mg/mL) once daily for seven consecutive days before tumour inoculation. On Day 0, mice were inoculated subcutaneously with ID8 cells, followed by a 4‐week period to allow tumour establishment. During this 4‐week period, the mice received AmB‐supplemented drinking water (0.5 μg/mL). The mice then received intratumoural injection of EBOT‐derived intratumoural microbiota once every 3 days for 4 weeks, while normal drinking water was maintained throughout the treatment period. (4) ET group: Mice received oral gavage of AmB (0.1 mg/mL) once daily for seven consecutive days before tumour inoculation. On Day 0, mice were inoculated subcutaneously with ID8 cells, followed by a 4‐week period to allow tumour establishment. During this 4‐week period, the mice received AmB‐supplemented drinking water (0.5 μg/mL). The mice then received intratumoural injection of EOC‐derived intratumoural microbiota once every 3 days for 4 weeks, while normal drinking water was maintained throughout the treatment period.

#### 2.5.2 Experiment 2

Effect of intratumoural *M. restricta* injection on tumour progression. A total of 27 female C57BL/6 mice were randomly divided into three groups (*n* = 9 per group): (1) M group: Mice were inoculated subcutaneously with ID8 cells on Day 0, followed by a 4‐week period to allow tumour establishment. The mice then received intratumoural injection of PBS once every 3 days for 4 weeks. (2) MT group: Mice were inoculated subcutaneously with ID8 cells on Day 0, followed by a 4‐week period to allow tumour establishment. The mice then received intratumoural injection of *M. restricta* suspension (1 × 10^5^ CFU/100 μL) once every 3 days for 4 weeks. (3) ET group: Mice were inoculated subcutaneously with ID8 cells on day 0, followed by a 4‐week period to allow tumour establishment. The mice then received intratumoural injection of EOC‐derived intratumoural microbiota once every 3 days for 4 weeks.

#### 2.5.3 Experiment 3

Effect of JAK2/STAT3 inhibition on *M. restricta*‐mediated tumour promotion. A total of 27 female C57BL/6 mice were randomly divided into three groups (*n* = 9 per group): (1) M group: Mice were inoculated subcutaneously with ID8 cells on Day 0, followed by a 4‐week period to allow tumour establishment. The mice then received intratumoural injection of PBS once every 3 days for 4 weeks. (2) MT group: Mice were inoculated subcutaneously with ID8 cells on Day 0, followed by a 4‐week period to allow tumour establishment. The mice then received intratumoural injection of *M. restricta* suspension (1 × 10^5^ CFU/100 μL) once every 3 days for 4 weeks. (3) MA group: Mice were inoculated subcutaneously with ID8 cells on Day 0, followed by a 4‐week period to allow tumour establishment. The mice then received intratumoural injection of *M. restricta* suspension (1 × 10^5^ CFU/100 μL) once every 3 days for 4 weeks, together with peritumoural injection of AG490 (10 mg/kg) during the same treatment period.

Tumour volume (1/2 × length × width^2^) and body weight were measured every 3 days. At the end of the experiment, blood and tumour tissues were collected. Tumours were weighed and either processed for histological analyses or stored at −80°C for subsequent molecular experiments.

### 2.6 Fungal Culture

*Malassezia restricta*, sourced from Beijing Biobw Biotechnology (China), was maintained on modified Dixon agar at 30°C. Fungi were subsequently expanded in liquid modified Dixon medium for experimental use.

### 2.7 Isolation and Characterisation of *Malassezia restricta*‐Derived Extracellular Vesicles

After three to four passages, a single colony of *M. restricta* was inoculated into modified Dixon liquid medium for pre‐culture and then expanded in fresh medium under shaking conditions for 48 h. The culture supernatants were sequentially centrifuged at 4000 × *g*, 10,000 × *g* and 15,000 × *g* at 4°C to remove cells and debris, concentrated using a 100 kDa Amicon ultrafiltration system, and filtered through a 0.45 μm polycarbonate membrane. EVs were then collected by ultracentrifugation at 100,000 × *g* for 1 h at 4°C using an Optima L‐100XP ultracentrifuge equipped with an SW 32 Ti rotor. The EV pellets were washed with pre‐cooled PBS and finally resuspended in 200 μL PBS. Samples were either used immediately for characterisation or stored at −80°C until use. EV morphology was examined by transmission electron microscopy (TEM) after negative staining with 2% phosphotungstic acid, and representative images were acquired at 80 kV. Particle concentration and size distribution were analysed by nanoparticle tracking analysis (NTA) using a ZetaView PMX 110 at 25°C.

### 2.8 Macrophage Uptake Assay

PKH26‐labelled MrEVs were prepared and incubated with RAW264.7 macrophages seeded on coverslips in 24‐well plates for 24 h at 37°C in a humidified incubator with 5% CO_2_. After incubation, the cells were washed with PBS, fixed with 4% paraformaldehyde, permeabilised with 0.3% Triton X‐100 and blocked with 5% bovine serum albumin. The cells were then incubated overnight at 4°C with an F4/80 antibody (1:100, 28463‐1‐AP, ProteinTech, China). F4/80 was used as a murine macrophage marker to identify RAW264.7 cells and outline their cell boundaries during confocal imaging, thereby facilitating visualisation of the uptake and intracellular distribution of PKH26‐labelled MrEVs. After incubation with the appropriate secondary antibody and DAPI nuclear staining, the coverslips were mounted and observed using a Zeiss LSM710 confocal microscope.

### 2.9 Cell Proliferation and Migration Assays

To assess the effects of macrophage‐derived conditioned media on tumour cell behaviour, ID8 cells were cultured with conditioned media (CM) collected from different macrophage treatment groups. Cell proliferation was evaluated using a CCK‐8 kit (Cat# C0038, Beyotime, China) according to the manufacturer's instructions. Briefly, ID8 cells were seeded into 96‐well plates and incubated with the indicated conditioned media, and the absorbance at 450 nm was measured using a microplate reader. For the wound‐healing assay, ID8 cells were seeded into 6‐well plates and grown to confluence. A linear scratch was made using a sterile pipette tip, and the cells were then cultured with the indicated conditioned media. Images were captured at 0, 12 and 24 h, and the migration rate was quantified using ImageJ software.

### 2.10 Haematoxylin and Eosin (H&E) Staining

Following initial fixation in 10% neutral buffered formalin, tumour specimens were encapsulated in paraffin to facilitate the generation of 2‐μm‐thick sections for H&E staining. Paraffin slices were dehydrated through ethanol, which were in turn immersed in xylene for two times, with 10 min each. Sections were stained with haematoxylin for 5 min, rinsed in water for 1 min, and differentiated using hydrochloric acid–ethanol and eosin solutions. Following dehydration in ethanol and clearing with xylene (two changes, 10 min each), the slices were mounted with neutral gum and morphologically examined under an optical microscope.

### 2.11 Immunohistochemistry (IHC)

To evaluate the expression of Ki‐67 across groups, paraformaldehyde‐fixed tissue sections (5 μm) were deparaffinised and rehydrated. To neutralise endogenous peroxidase, sections were exposed to 3% H_2_O_2_ for 10 min. This was followed by a 1 h blocking step at room temperature using 5% goat serum to prevent non‐specific binding. The sections were then incubated with the primary antibody against Ki‐67 (1:1000, GB11499, Servicebio, China) at 4°C overnight. Following the rinsing steps, immunoreactivity was visualised by applying the secondary antibody. Ki‐67‐positive cells, characterised by brown nuclear staining, were observed and documented using a bright‐field microscope. For quantitative analysis, three randomly selected high‐power fields from each section were analysed, and the Ki‐67 index was calculated as the percentage of Ki‐67‐positive nuclei relative to the total number of nuclei.

### 2.12 Immunofluorescence (IF) Staining

Following routine dewaxing and thermal‐induced antigen unmasking, tissue sections underwent 30 min of blocking with 3% BSA at ambient temperature. The specimens were then incubated overnight at 4°C with primary antibodies against CD86 (1:100, A16805, ABclonal), CD206 (1:100, A21014, ABclonal), CD8a (1:100, 29896‐1‐AP, Proteintech) and CD4 (1:100, A0362, ABclonal). After three 5‐min washes with PBS, the signals were developed using appropriate fluorophore‐conjugated secondary antibodies (2 h, room temperature) in the dark. Nuclear counterstaining was achieved using DAPI for a 5‐min period. This was followed by embedding in 10% glycerol, after which the fluorescence signals were detected and imaged using a fluorescence microscope.

### 2.13 Fluorescence In Situ Hybridisation (FISH) Analysis

Fluorescence in situ hybridisation (FISH) was performed on formalin‐fixed, paraffin‐embedded (FFPE) tissue sections from EBOT and EOC tissues to detect intratumoural fungal signals. Briefly, tissue sections were washed in 0.1 M Tris–HCl buffer (pH 7.4) for 15 min, followed by enzymatic treatment with lysozyme (10 mg/mL) for 30 min to facilitate fungal cell wall digestion. The sections were then hybridised with the pan‐fungal oligonucleotide probe D223 targeting fungal 28S rRNA (5′‐CCACCCACTTAGAGCTGC‐3′), labelled with a 5′‐Cy3 fluorophore (excitation: 555 nm; emission: 570 nm; Molecular Probes). Hybridisation signals were observed and imaged using a Pannoramic 250/MIDI scanner.

### 2.14 Reverse Transcription Quantitative PCR (RT‐qPCR)

Isolation of total RNA from both tumour specimens and cell cultures was achieved with TRIzol (Invitrogen, USA). Subsequent cDNA synthesis was carried out utilising the PrimeScript RT Master Mix (Takara Bio, Japan). To quantify gene expression, Real‐time PCR was executed via SYBR Green chemistry on an ABI ViiA 7 platform (Applied Biosystems, USA). Data normalisation was performed against β‐actin, with the 2^−ΔΔCt^ algorithm employed for relative quantification. Primers used in this study are listed in Table S3.

### 2.15 Western Blot

Following denaturation, protein extracts underwent electrophoretic separation on 8%–15% SDS‐PAGE gradients. The resulting protein bands were immobilised onto PVDF membranes (Millipore, IPVH00010) via a wet‐transfer system. To minimise non‐specific signals, the membranes were equilibrated in a 5% non‐fat milk/TBS‐T solution for 120 min at room temperature. After thorough rinsing with TBS‐T (three 5‐min cycles), overnight incubation with primary antibodies (detailed in Table S4) was performed at 4°C. After 10‐min TBS‐T washes for three times, secondary antibodies that were horseradish peroxidase (HRP)‐conjugated and species‐matched were maintained at ambient temperature for 1 h. Subsequent to triple 10‐min rinses in TBS‐T, the membranes were subjected to chemiluminescent visualisation using the SuperSignal West Pico PLUS kit (Thermo Scientific, 34577). The resulting protein bands were documented with a ChemiDoc MP Imaging System (Bio‐Rad), utilising auto‐optimised exposure parameters for signal acquisition.

### 2.16 Enzyme‐Linked Immunosorbent Assay (ELISA)

The levels of IL‐6, TNF‐α, IL‐10 and TGF‐β in cell culture supernatants and tumour tissue homogenates were measured using ELISA kits (IL‐6, Cat# EK206; TNF‐α, Cat# EK282; IL‐10, Cat# EK210; TGF‐β, Cat# EK981) purchased from MultiSciences Biotech (Hangzhou, China), following the manufacturer's instructions. Briefly, cell culture supernatants and tumour tissue homogenates were collected, and standards and samples were added to the corresponding wells. After incubation with detection reagents, colour development was performed, and the absorbance was measured at 450 nm using a microplate reader. Cytokine concentrations were calculated based on the respective standard curves.

### 2.17 Fungal DNA Extraction and Sequencing

DNA was extracted from tumour tissues using commercial kits (Omega M5636‐02; Magen D6356‐03). Following quantification and purity verification via a NanoDrop 2000 spectrophotometer, the samples were archived at −20°C. The ITS1 rDNA segment was targeted for amplification using the specific primers 5′‐CTTGGTCATTTAGAGGAAGTAA‐3′ (Forward) and 5′‐GCTGCGTTCTTCATCGATGC‐3′ (Reverse), with subsequent sequencing executed on an Illumina NovaSeq instrument. The ITS1 rDNA sequencing data generated in this study have been deposited in the NCBI Sequence Read Archive (SRA) under BioProject accession number PRJNA1369007 (https://www.ncbi.nlm.nih.gov/).

### 2.18 Statistical Analysis

Data distribution was first evaluated for normality through the Shapiro–Wilk test. For variables following a normal distribution, statistical differences were determined via unpaired *t*‐tests (two groups) or one‐way ANOVA (multiple groups). In cases of non‐normally distributed data, the Mann–Whitney *U* or Wilcoxon signed‐rank tests were employed for independent or paired comparisons, respectively. Values are expressed as mean ± SEM or SD, with a significance threshold set at *p* < 0.05.

## 3 Results

### 3.1 Intratumoural Mycobiome Dysbiosis in Patients With EOC

To examine the potential influence of the intratumoural microbiota on EOC development, we recruited a cohort of 20 patients with EOC and 20 patients with EBOT. All participants were of Asian descent, with ages ranging from 18 to 75 years. Age and body mass index (BMI) remained comparable between the two cohorts, with no discernible statistical variation (*p* > 0.05). The cohorts were also well matched for key lifestyle and reproductive factors, including smoking history, menstrual and obstetric background, tubal ligation status, and use of oral contraceptives. As expected, however, the EOC group exhibited significantly higher serum CA125 levels than the EBOT group (*p* < 0.001) (Table S5).

**FIGURE 1:**
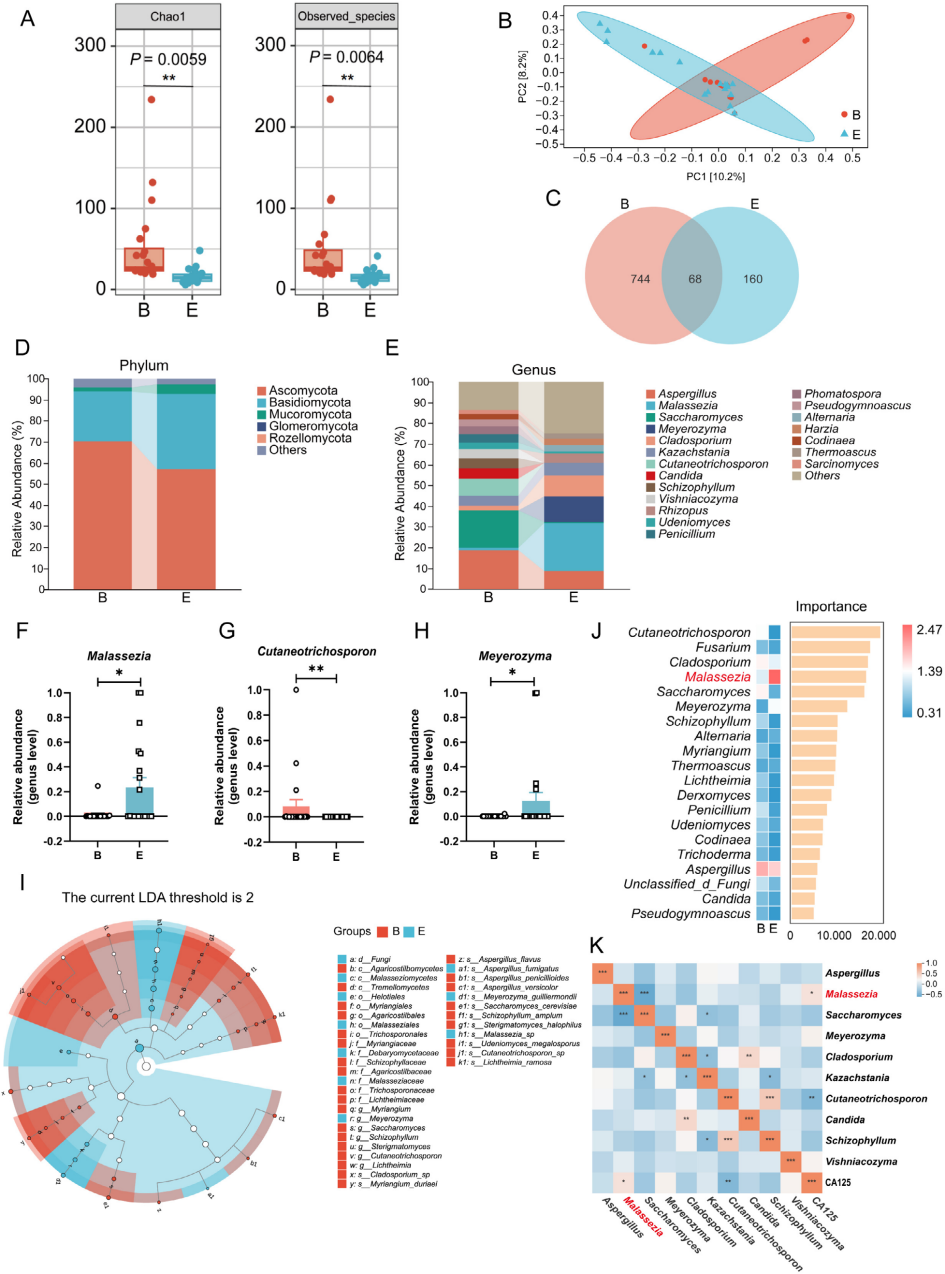
Intratumoural mycobiome dysbiosis in patients with EOC. (A) Alpha‐diversity analysis. (B) Beta‐diversity analysis. (C) Venn diagram. (D, E) Distribution of fungal communities at both phylum and genus levels. (F–H) Comparative abundance of *Malassezia*, *Cutaneotrichosporon* and *Meyerozyma*. (I) Differential fungal taxa identified by LEfSe (LDA score > 2). (J) Random Forest analysis. (K) Correlation matrix (Heatmap) linking intratumoral mycobiota with serum CA125 levels; colour gradients represent positive (red) and negative (blue) associations. B: EBOT group (*n* = 20); E: EOC group (*n* = 20). Data are presented as mean ± SEM. *p < 0.05, **p < 0.01.

To provide spatial evidence for the presence of intratumoural fungi in EBOT and EOC tissues, we performed FISH, which detected fungal signals in both groups, with stronger signals observed in EOC tissues than in EBOT tissues (Figure S1A). We next performed ITS1 rDNA sequencing on tumour samples to investigate alterations in the intratumoural fungal landscape. The EOC group showed a marked reduction in α‐diversity compared with the EBOT group, as reflected by significantly lower Chao1 indices (*p* = 0.0059) and observed species counts (*p* = 0.0064) (Figure 1A). Principal coordinate analysis of β‐diversity further demonstrated clear separation between the two cohorts, indicating substantial differences in overall fungal community structure (Figure 1B). Consistent with these findings, Venn diagram analysis identified 812 and 228 unique amplicon sequence variants in the EBOT and EOC groups, respectively, further supporting reduced fungal diversity in malignant tissue (Figure 1C). At the phylum level, Ascomycota and Basidiomycota were the dominant taxa in both groups (Figure 1D). By contrast, genus‐level taxonomic profiling revealed pronounced compositional differences (Figure 1E). Specifically, the EOC mycobiome was characterised by a significant enrichment of *Malassezia* and *Meyerozyma*, alongside a marked depletion of *Cutaneotrichosporon* compared with the EBOT group (Figure 1F–H). Both linear discriminant analysis effect size and random forest analyses consistently identified *Malassezia* and *Meyerozyma* as key taxonomic features of EOC, whereas *Cutaneotrichosporon* was strongly associated with EBOT samples (Figure 1I,J). To further validate the ITS1 sequencing results, we performed *Malassezia*‐specific qPCR, which confirmed the increased abundance of *Malassezia* in EOC tissues compared with EBOT tissues (Figure S1B). In addition, correlation analysis demonstrated a positive association between *Malassezia* abundance and the clinical tumour marker CA125 (Figure 1K). Together, these results indicate pronounced dysbiosis of the intratumoural mycobiome in EOC and identify *Malassezia* as a prominent resident fungus associated with disease progression.

### 3.2 Human Epithelial Ovarian Carcinoma Intratumoural Microbiota Promote EOC Progression in Mice

**FIGURE 2:**
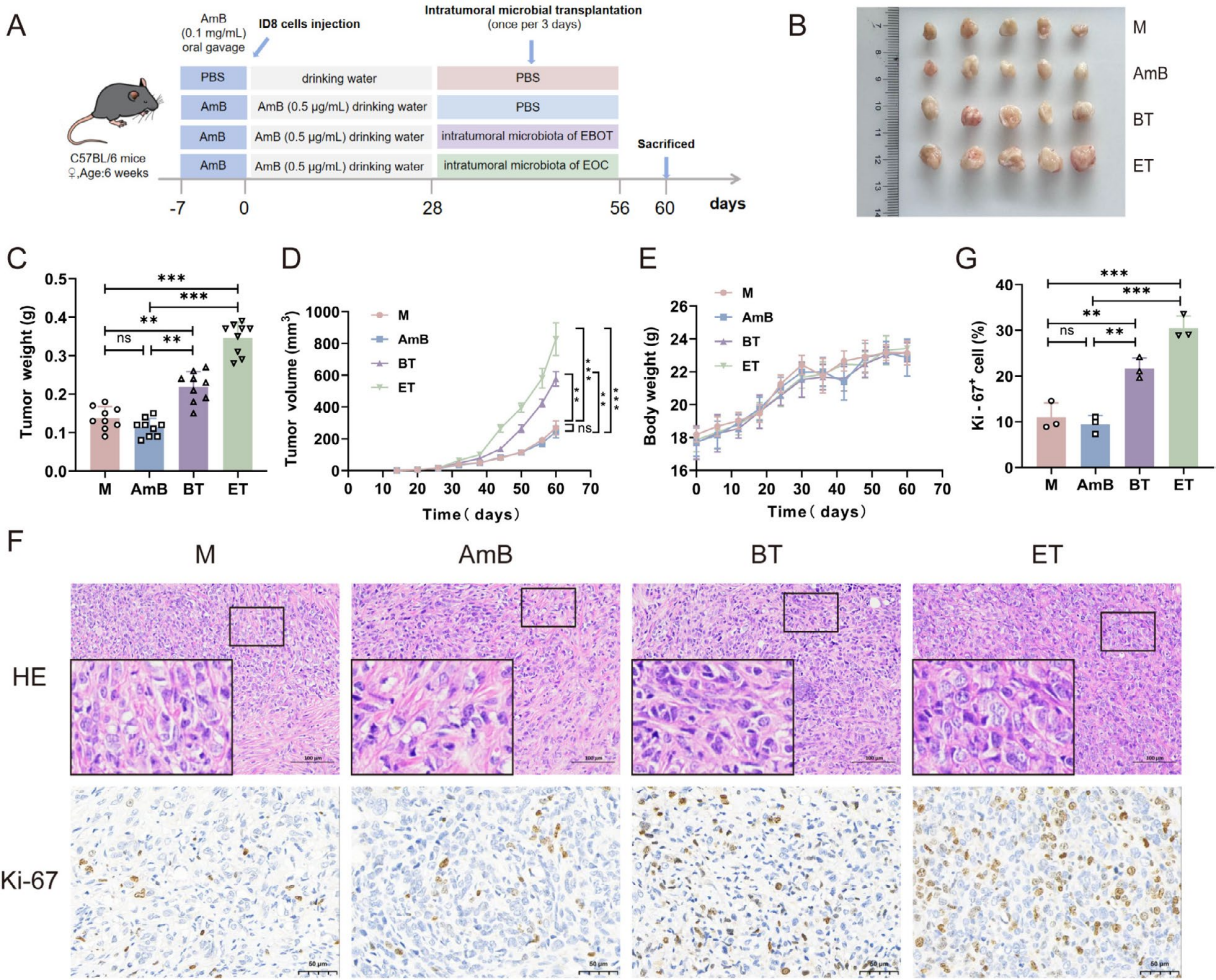
Human epithelial ovarian carcinoma intratumoural microbiota promote EOC progression in mice. (A) Experimental design schematic. (B) Representative photographs of tumours harvested on Day 60 across various intervention groups. (C) Tumour weight of mice in the M, AmB, BT and ET groups on Day 60 (*n* = 9). (D) Tumour volume changes over time in the M, AmB, BT and ET groups (*n* = 9). (E) Body weight changes of mice over time in the M, AmB, BT and ET groups (*n* = 9). (F) H&E and IHC staining of tumour tissues. Scale bars: 100 and 50 μm. (G) Quantification of Ki‐67^+^ cells in each group. Groups: M (ID8 tumour‐bearing model group); AmB (amphotericin B‐treated); BT (EBOT‐derived microbial transfer); ET (EOC‐derived microbial transfer). Data are presented as mean ± SD. ns, not significant; **p < 0.01, ***p < 0.001.

To assess the contribution of the intratumoural microbiota to EOC progression, we established a murine transplantation model. Mice were first treated with AmB by oral gavage for seven consecutive days, followed by continuous supplementation in drinking water for 4 weeks, to deplete endogenous fungal populations. Intratumoural microbiota harvested from patients with either EBOT or EOC were then transplanted into tumour‐bearing mice by direct intratumoural injection (Figure 2A). Tumour growth was monitored longitudinally. No significant differences in tumour volume or weight were observed between the control (M group) and fungal‐depleted (AmB group) mice (Figure 2B–D), indicating that fungal depletion alone does not affect tumour growth dynamics. By contrast, mice receiving microbiota from either EBOT (BT group) or EOC (ET group) patients displayed markedly accelerated tumour growth, with a substantially increased tumour burden compared with the M and AmB groups (Figure 2B–D). Notably, body weights remained stable and comparable across all experimental groups (Figure 2E). Histological analysis using H&E staining revealed dense clusters of neoplastic cells with pronounced nuclear atypia in both the BT and ET groups, with more severe pathological changes observed in the ET group. Consistent with these findings, IHC staining for Ki‐67 demonstrated increased cellular proliferation in the BT and ET groups compared with the M group, and quantitative analysis further confirmed that the proportion of Ki‐67‐positive cells was significantly increased in the BT and ET groups, with a more pronounced elevation in the ET group (Figure 2F,G). Together, these results indicate that intratumoural microbiota promote EOC progression in vivo.

### 3.3 *Malassezia restricta* Colonisation Promotes EOC Progression and Enhances M2 Macrophage Infiltration in the TME

**FIGURE 3:**
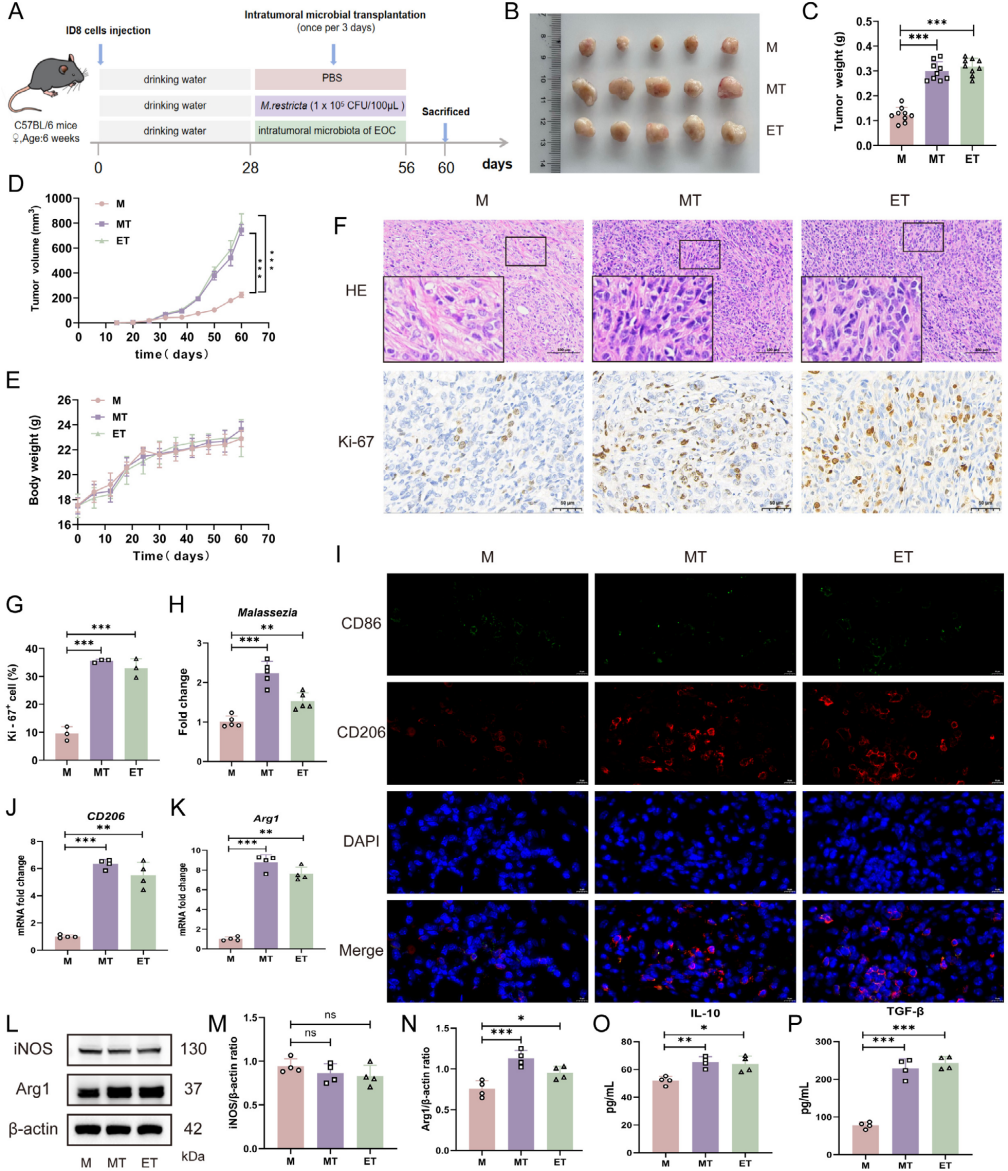
*Malassezia restricta* colonisation promotes EOC progression and enhances M2 macrophage infiltration in the TME. (A) Experimental design schematic. (B) Tumour images from mice with different treatments on Day 60. (C) Tumour weight in the M, MT and ET groups on Day 60 (*n* = 9). (D) Tumour volume changes over time in the M, MT and ET groups (*n* = 9). (E) Body weight changes of mice in the M, MT and ET groups over time (*n* = 9). (F) H&E and IHC staining of tumour tissues. Scale bars: 100 and 50 μm. (G) Quantification of Ki‐67^+^ cells in each group. (H) Relative abundance of *Malassezia* determined by qPCR (*n* = 5). (I) IF staining for CD86 and CD206. Scale bar: 10 μm. (J, K) mRNA levels of *CD206* and *Arg1* (*n* = 4). (L–N) Protein levels of iNOS and Arg1 (*n* = 4). (O, P) Levels of IL‐10, TGF‐β (*n* = 4). Groups: M (ID8 tumour‐bearing model group); MT (*M. restricta* colonisation); ET (EOC‐derived microbial transfer). Data are presented as mean ± SD. ns, not significant; *p < 0.05, **p < 0.01, ***p < 0.001.

Given that *M. restricta* is among the most prevalent fungal species identified across diverse tumour types (Narunsky‐Haziza et al. 2022), we hypothesised that it may actively drive EOC progression. To assess the in vivo effects of *M. restricta* on tumour growth, a subcutaneous EOC mouse model was established (Figure 3A). Mice challenged with *M. restricta* (MT group) exhibited significantly accelerated tumour growth compared with the control group (M group), as evidenced by markedly increased tumour volumes and weights (Figure 3B–D). Body weights remained stable and comparable across all groups, indicating the absence of overt systemic toxicity (Figure 3E). Histological analysis by H&E staining revealed dense neoplastic cell populations with pronounced nuclear atypia in both the MT and ET groups (Figure 3F). Furthermore, IHC staining for Ki‐67 confirmed enhanced cellular proliferation, and quantitative analysis further showed that the proportion of Ki‐67‐positive cells was significantly increased in the ET and MT groups (Figure 3F,G). To confirm successful *M. restricta* colonisation within the tumour tissue, its abundance was quantified by qPCR. The MT and ET groups showed significantly higher levels of *M. restricta* than the M group (Figure 3H). Together, these results indicate that intratumoural colonisation by *M. restricta* markedly accelerates EOC progression.

Microbes are known to promote oncogenesis by modulating the TME (Xu, Ding, et al. 2025; Xu, Luo, et al. 2025; Xu, Qiao, et al. 2025; Zhang, Feng, et al. 2025; Zhang, Huang, et al. 2025; Zhang, Xiang, et al. 2025; Zhang, You, et al. 2025). To investigate how *M. restricta* shapes the TME, IF staining was performed to assess the infiltration of immune cell subsets, including M1 and M2 macrophages, as well as CD4^+^ and CD8^+^ T cells, in murine tumour tissues. Tumours colonised by *M. restricta* showed a significant increase in CD206^+^ M2 macrophages compared with the control group. By contrast, the proportions of CD86^+^ M1 macrophages and CD4^+^ and CD8^+^ T cells were largely unchanged across groups (Figure 3I, Figure S2A). These observations were further supported by RT‐qPCR analysis, which revealed marked upregulation of M2‐associated markers (CD206 and Arg1) in the MT group, whereas M1‐associated markers (CD86 and iNOS) did not differ significantly (Figure 3J,K, Figure S2B,C). Similarly, the expression of genes related to CD4^+^ and CD8^+^ T‐cell function showed no significant differences between groups (Figure S2D–G). In line with these findings, western blot analysis demonstrated increased Arg1 protein expression in tumours from the MT group, while iNOS levels remained unchanged (Figure 3L–N). Moreover, ELISA results showed significantly elevated levels of M2‐associated cytokines, including IL‐10 and TGF‐β, in the MT group (Figure 3O,P). By contrast, levels of M1‐associated cytokines, such as IL‐6 and TNF‐α, were not significantly altered (Figure S2H,I). Collectively, these data indicate that *M. restricta* promotes EOC progression by enhancing M2 macrophage infiltration within the TME.

### 3.4 Soluble Components of *Malassezia restricta* Drive Macrophage M2 Polarisation

**FIGURE 4:**
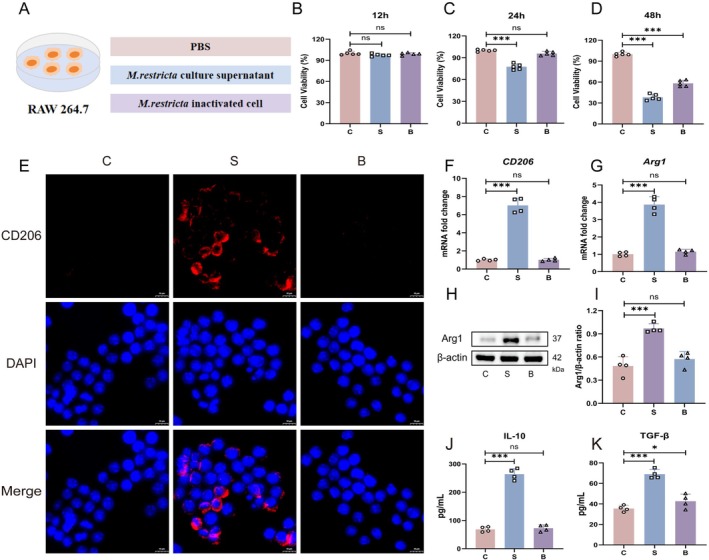
Soluble components of *Malassezia restricta* drive macrophage M2 polarisation. (A) Flow chart of the cell experiment design. (B–D) Cell viability was assessed using the CCK‐8 assay (*n* = 5). (E) IF staining for CD206. Scale bar: 10 μm. (F, G) mRNA levels of *CD206* and *Arg1* (*n* = 4). (H, I) Protein levels of Arg1 (*n* = 4). (J, K) Levels of IL‐10, TGF‐β (*n* = 4). Groups: C (control group); S (treated with *M. restricta* culture supernatant for 24 h); B (treated with heat‐inactivated *M. restricta* cell bodies for 24 h). Data are presented as mean ± SD. ns, not significant; ***p < 0.001.

To identify the fungal components responsible for inducing M2 macrophage polarisation, the culture supernatant and heat‐inactivated cell bodies of *M. restricta* were isolated by centrifugation and co‐incubated with RAW264.7 cells (Figure 4A). A CCK‐8 assay was used to assess RAW264.7 cell viability following exposure to these components. No significant difference in cell viability was observed after 12 h of treatment. At 24 h, the culture supernatant significantly reduced RAW264.7 cell viability, whereas the heat‐inactivated cell bodies showed no significant effect. After 48 h of treatment, both the culture supernatant and the heat‐inactivated cell bodies reduced cell viability, with the supernatant showing a more pronounced effect (Figure 4B–D). Since the reduction in viability was more substantial after 48 h of exposure, 24 h was selected as the working time point to observe fungal component‐induced macrophage responses while avoiding the more severe viability loss observed after prolonged exposure. Notably, IF staining for CD206 showed that the culture supernatant, rather than the cell bodies, effectively induced M2 macrophage polarisation (Figure 4E). This observation was further supported by RT‐qPCR analysis, which demonstrated that treatment with the supernatant markedly increased the mRNA expression of CD206 and Arg1 (Figure 4F,G). Consistent with these findings, western blot analysis revealed a significant increase in Arg1 protein levels in macrophages exposed to the supernatant (Figure 4H,I). In addition, ELISA assays confirmed elevated levels of IL‐10 and TGF‐β in the RAW264.7 cell culture following supernatant treatment (Figure 4J,K). Taken together, these results indicate that the *M. restricta* culture supernatant contains key bioactive components that drive M2 macrophage differentiation.

### 3.5 EVs From *Malassezia restricta* Are Internalised by Macrophages and Promote M2 Polarisation

**FIGURE 5:**
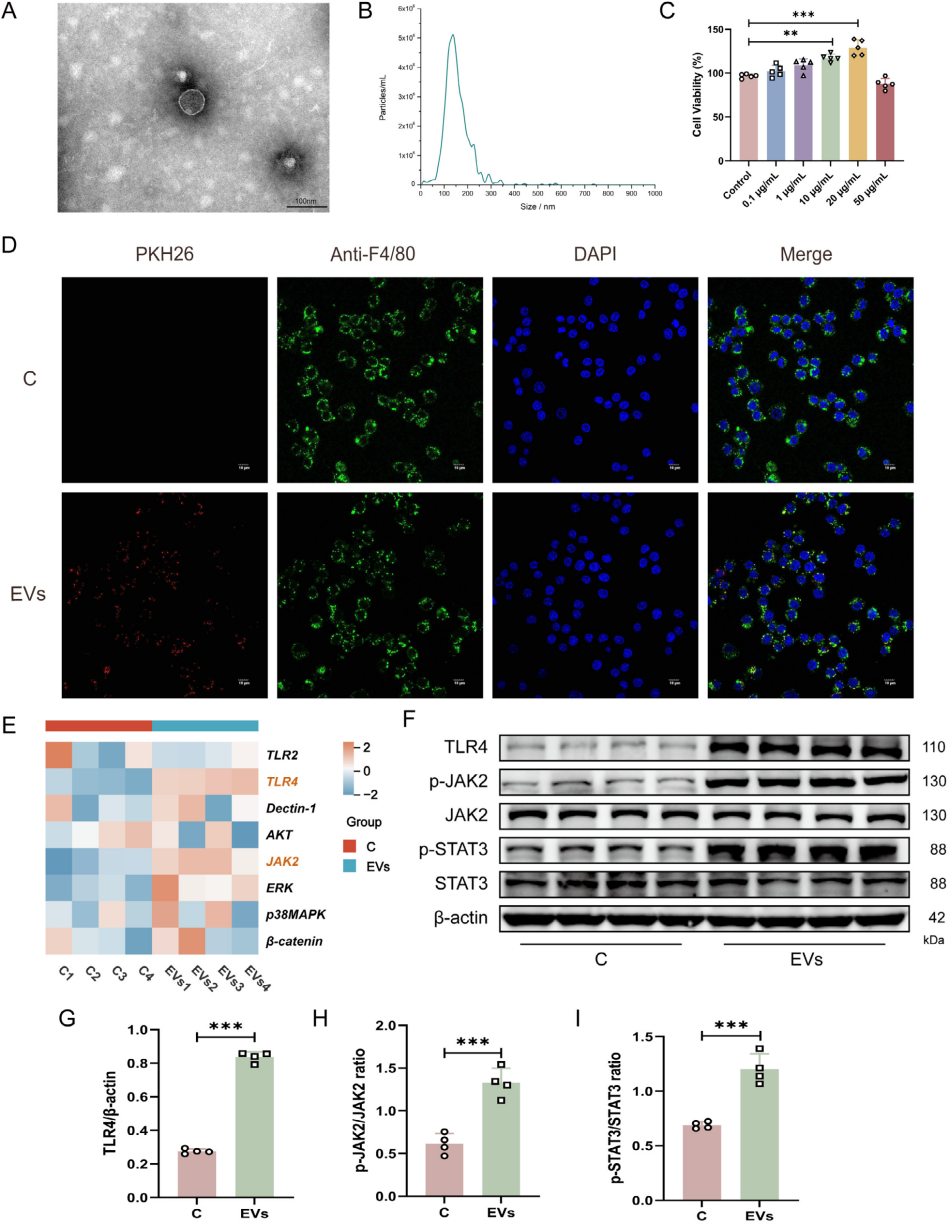
EVs from *Malassezia restricta* are internalised by macrophages and promote M2 polarisation. (A) TEM image of MrEVs. Scale bars, 100 nm. (B) NTA analysis of MrEVs. (C) Cell viability was assessed using the CCK‐8 assay (*n* = 5). (D) Fluorescent pictures of MrEVs endocytosed by RAW 264.7 cells. Scale bars, 10 μm. (E) Relative mRNA expression levels of signalling pathways associated with macrophage M2 polarisation in different treatment groups (*n* = 4). (F–I) Protein levels of TLR4, phosphorylated JAK2 (p‐JAK2), total JAK2, phosphorylated STAT3 (p‐STAT3), and total STAT3 following MrEVs stimulation (*n* = 4). Groups: C (control group); EVs (treated with MrEVs at 20 μg/mL for 24 h). Data are presented as mean ± SD. **p < 0.01, ***p < 0.001.

Previous studies have shown that microbe‐derived EVs act as potent modulators of macrophage activity (Qu et al. 2022; Bhanu et al. 2025). We therefore hypothesised that EVs secreted by *M. restricta* (MrEVs) play a central role in driving M2 macrophage polarisation. To test this hypothesis, MrEVs were isolated from the culture supernatant by differential ultracentrifugation. TEM revealed that purified MrEVs displayed a characteristic cup‐shaped or spherical morphology with a distinct bilayer membrane structure (Figure 5A). Nanoparticle tracking analysis showed that the vesicles ranged from 50 to 300 nm in diameter, with an average size of 154.8 nm (Figure 5B). To assess the effects of MrEVs on macrophage viability, RAW264.7 cells were treated with increasing concentrations of MrEVs and analysed using a CCK‐8 assay. MrEVs increased macrophage viability at concentrations ranging from 0.1 to 20 μg/mL. At 50 μg/mL, a slight numerical decrease in the CCK‐8 signal was observed; however, this change was not statistically significant. Therefore, 20 μg/mL was selected as the optimal working concentration for subsequent experiments (Figure 5C). To visualise MrEV internalisation, vesicles were labelled with PKH26 and incubated with RAW264.7 cells. Confocal laser scanning microscopy confirmed efficient uptake of MrEVs within 24 h, with predominant accumulation in the perinuclear region of macrophages (Figure 5D). These results indicate that MrEVs are actively internalised by macrophages and may function as key mediators of macrophage phenotypic modulation.

To further confirm the regulatory effects of MrEVs on macrophage activation, a series of functional assays was performed using RAW264.7 cells (Figure S3A). IF staining showed a marked increase in CD206 expression following MrEV exposure (Figure S3B). In line with this, RT‐qPCR analysis demonstrated robust upregulation of CD206 and Arg1 mRNA levels in MrEV‐treated macrophages compared with untreated controls (Figure S3C,D). Western blot analysis further supported these findings, revealing significantly increased Arg1 protein expression in the MrEV‐treated group (Figure S3E,F). In addition, ELISA results showed that MrEV treatment significantly enhanced the secretion of M2‐associated cytokines, including IL‐10 and TGF‐β (Figure S3G,H). Together, these data indicate that MrEVs largely recapitulate the immunomodulatory effects of the *M. restricta* culture supernatant and represent a principal driver of M2 macrophage polarisation.

To elucidate the molecular mechanisms underlying MrEV‐induced macrophage activation, a series of mechanistic analyses was conducted. Because Dectin‐1, TLR2 and TLR4 are key receptors involved in fungal pathogen recognition (Plato et al. 2015; Kaminski et al. 2024), we hypothesised that MrEVs engage macrophages through specific pattern recognition receptors, thereby initiating downstream signalling pathways that promote M2 polarisation. We first examined the expression of several fungal pattern recognition receptors, including Dectin‐1, TLR2 and TLR4. RT‐qPCR analysis showed that TLR4 exhibited the most pronounced upregulation following MrEV treatment. We further assessed the expression of genes related to M2 macrophage polarisation, including AKT, JAK2, ERK, p38MAPK and β‐catenin. Compared with the control group, MrEV‐treated macrophages showed a significant increase in JAK2 mRNA expression, whereas no significant changes were observed in the other genes examined (Figure 5E, Figure S4). Consistent with these transcriptional changes, western blot analysis confirmed that MrEV exposure markedly increased TLR4 protein expression and significantly enhanced phosphorylation of JAK2 and STAT3 (Figure 5F–I). Collectively, these findings indicate that MrEVs promote M2 macrophage polarisation through activation of the TLR4/JAK2/STAT3 signalling axis.

### 3.6 MrEVs Induce Macrophage M2 Polarisation via the JAK2/STAT3 Pathway to Promote the Proliferation of Ovarian Cancer Cells In Vitro

**FIGURE 6:**
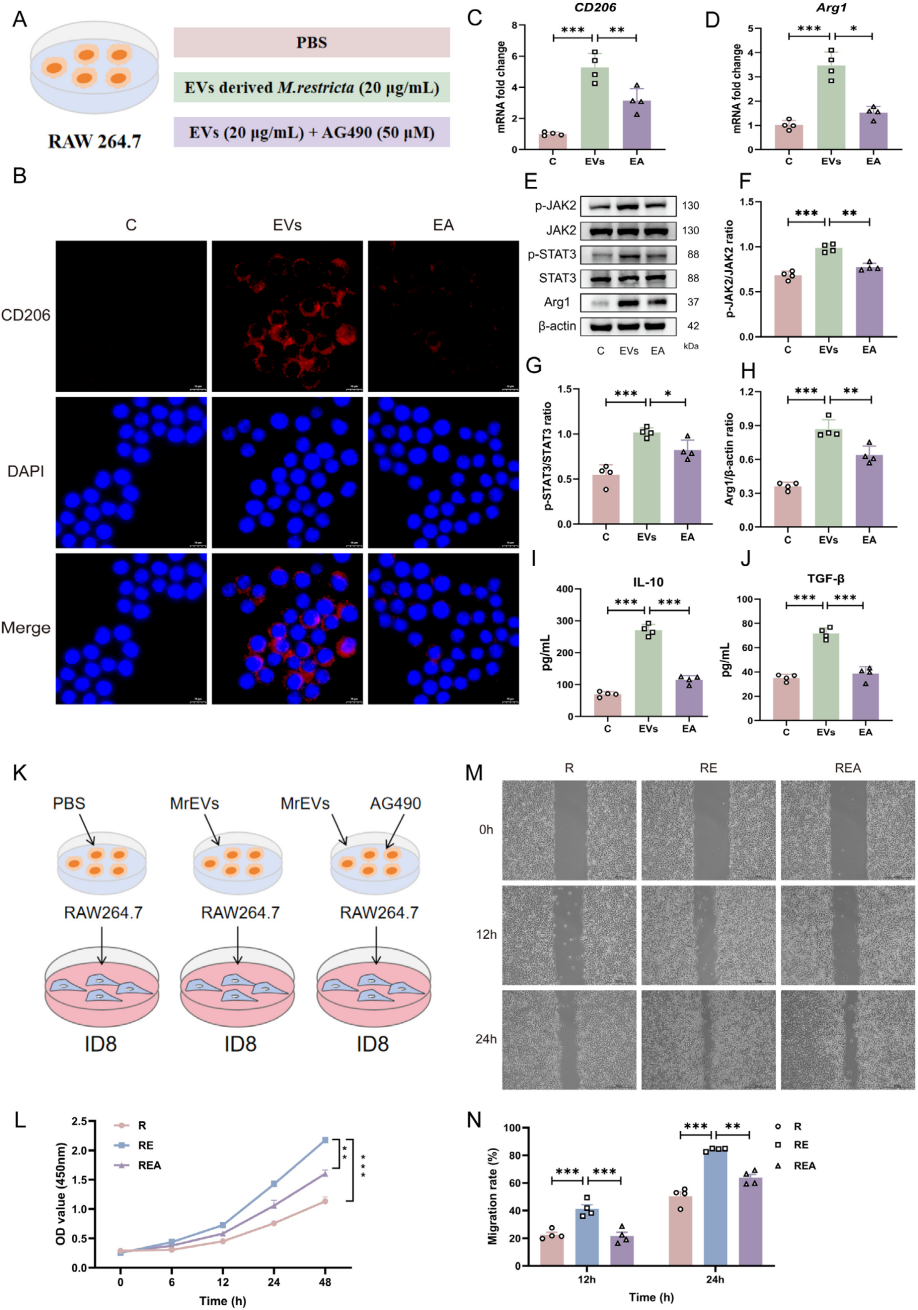
MrEVs induce macrophage M2 polarisation via the JAK2/STAT3 pathway to promote the proliferation of ovarian cancer cells in vitro. (A) Diagram of the inhibitor intervention experiment. (B) IF staining for CD206. Scale bar: 10 μm. (C, D) mRNA levels of *CD206* and *Arg1* (*n* = 4). (E–H) Protein levels of p‐JAK2, JAK2, p‐STAT3, STAT3 and Arg1 (*n* = 4). (I, J) levels of IL‐10, TGF‐β (*n* = 4). Groups: C (control group); EVs (MrEVs treatment for 24 h); EA (pretreated with AG490 for 4 h, followed by MrEVs treatment for 24 h). (K) Schematic diagram of the experimental procedure in which ID8 cells were treated with conditioned media derived from differently treated RAW264.7 macrophages. (L) CCK‐8 cell proliferation assay (*n* = 5). (M, N) Cell scratch assays (*n* = 4). Scale bar: 500 μm. Groups: R (ID8 cells treated with conditioned media from RAW264.7 macrophages); RE (ID8 cells treated with conditioned media from MrEV‐treated RAW264.7 macrophages); REA (ID8 cells treated with conditioned media from MrEV‐treated RAW264.7 macrophages in the presence of AG490). Data are presented as mean ± SD. *p < 0.05, **p < 0.01, ***p < 0.001.

To confirm that MrEVs promote M2 macrophage polarisation through the JAK2/STAT3 signalling pathway, the JAK2‐specific inhibitor AG490 was used (Figure 6A). RAW264.7 cells treated with MrEVs for 24 h showed a marked increase in the proportion of CD206^+^ M2 macrophages; this effect was significantly attenuated by co‐treatment with AG490 (Figure 6B). Consistent with these observations, RT‐qPCR analysis showed that AG490 significantly suppressed the MrEV‐induced upregulation of CD206 and Arg1 mRNA expression (Figure 6C,D). Western blot analysis further demonstrated that MrEV treatment increased Arg1 protein levels and enhanced phosphorylation of JAK2 and STAT3, effects that were effectively reversed by AG490 (Figure 6E–H). In addition, MrEV‐induced secretion of IL‐10 and TGF‐β was significantly reduced following pharmacological inhibition of JAK2 (Figure 6I,J). Together, these results confirm that MrEVs induce M2 macrophage polarisation through activation of the JAK2/STAT3 signalling cascade.

Accumulating evidence indicates that M2 macrophages promote tumour metastasis and invasion through paracrine secretion of multiple cytokines (Liu et al. 2020; Bied et al. 2023). To investigate macrophage–tumour cell crosstalk, ID8 cells were cultured with conditioned media derived from macrophages pretreated with MrEVs (Figure 6K). Under these conditions, MrEV‐educated macrophages significantly promoted ID8 cell proliferation, whereas this pro‐tumourigenic effect was markedly attenuated by AG490 treatment (Figure 6L). Similarly, wound‐healing assays showed that conditioned media from MrEV‐stimulated macrophages significantly enhanced the migratory capacity of ID8 cells, and this effect was effectively abolished by JAK2 inhibition with AG490 (Figure 6M,N). Collectively, these findings suggest that MrEVs promote M2 macrophage polarisation through activation of the JAK2/STAT3 signalling pathway, thereby enhancing the proliferation and migration of ovarian cancer cells.

### 3.7 *Malassezia restricta* Promotes EOC Progression In Vivo via JAK2/STAT3‐Mediated Macrophage M2 Polarisation

**FIGURE 7:**
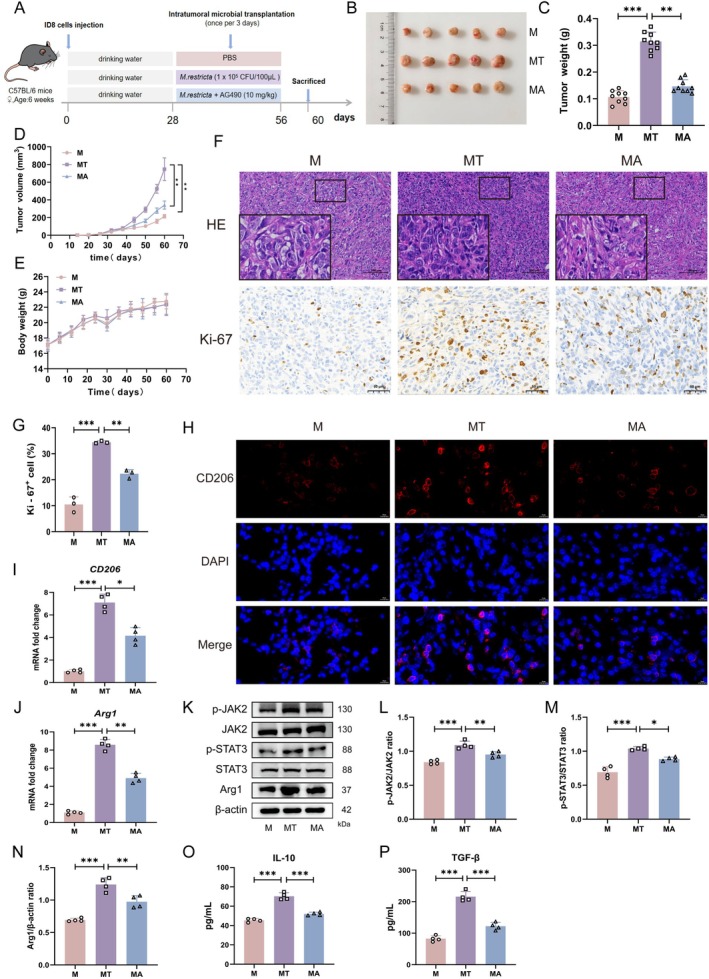
*Malassezia restricta* promotes EOC progression in vivo via JAK2/STAT3‐mediated macrophage M2 polarisation. (A) Diagram of the experimental design. (B) Tumour images from mice with different treatments on Day 60. (C) Tumour weight in the M, MT and MA groups on Day 60 (*n* = 9). (D) Tumour volume changes over time in the M, MT and MA groups (*n* = 9). (E) Body weight changes of mice in the M, MT and MA groups over time (*n* = 9). (F) H&E and IHC staining of tumour tissues. Scale bars: 100 and 50 μm. (G) Quantification of Ki‐67^+^ cells in each group. (H) IF staining for CD206. Scale bar: 10 μm. (I, J) mRNA levels of *CD206* and *Arg1* (*n* = 4). (K–N) Protein levels of p‐JAK2, JAK2, p‐STAT3, STAT3 and Arg1 (*n* = 4). (O, P) Levels of IL‐10, TGF‐β (*n* = 4). Groups: M (ID8 tumour‐bearing model group); MT (*M. restricta* colonisation); MA (*M. restricta* colonisation and AG490 treatment). Data are presented as mean ± SD. *p < 0.05, **p < 0.01, ***p < 0.001.

To determine whether *M. restricta* promotes EOC progression through the JAK2/STAT3 signalling axis in vivo, three experimental cohorts were established: an EOC control group (M group), an *M. restricta* colonisation group (MT group), and an AG490 intervention group (MA group), in which mice colonised with *M. restricta* were treated with the JAK2 inhibitor AG490 (Figure 7A). In line with previous findings, *M. restricta* colonisation markedly accelerated tumour growth. Importantly, AG490 administration effectively abrogated this pro‐tumourigenic effect, as demonstrated by significant reductions in both tumour volume and weight compared with the MT group (Figure 7B–D). No significant differences in body weight were observed among the three groups, indicating good tolerability of AG490 treatment (Figure 7E). Histological examination by H&E staining showed reduced cellular density and attenuated nuclear pleomorphism in tumours from the MA group relative to the MT group, consistent with decreased tumour malignancy (Figure 7F). Concordantly, Ki‐67 staining revealed a marked reduction in proliferative activity in the MA group, and quantitative analysis further confirmed that the proportion of Ki‐67‐positive cells was significantly decreased, indicating that JAK2 inhibition suppressed the hyperproliferative phenotype induced by *M. restricta* (Figure 7F,G). Mechanistically, IF analysis demonstrated that AG490 treatment effectively blocked the infiltration of CD206^+^ M2 macrophages in tumour tissues (Figure 7H). These findings were further supported by RT‐qPCR and western blot analyses, which showed that AG490 significantly reduced the expression of M2‐associated markers (Arg1 and CD206) and inhibited phosphorylation of JAK2 and STAT3 (Figure 7I–N). In addition, ELISA results confirmed that the elevated levels of IL‐10 and TGF‐β induced by *M. restricta* colonisation were significantly decreased following AG490 treatment (Figure 7O,P). Together, these in vivo rescue experiments demonstrate that *M. restricta* promotes EOC progression by driving M2 macrophage polarisation through activation of the JAK2/STAT3 signalling pathway.

## 4 Discussion

EOC remains one of the deadliest gynaecological malignancies (Lheureux, Braunstein, and Oza 2019; Lheureux, Gourley, et al. 2019; Kuroki and Guntupalli 2020). Emerging evidence indicates that tumour‐resident fungi actively remodel the TME, thereby facilitating cancer progression (Saftien et al. 2023; Galloway‐Peña et al. 2024; Zhang, Feng, et al. 2025; Zhang, Huang, et al. 2025; Zhang, Xiang, et al. 2025; Zhang, You, et al. 2025). However, the diversity, biological functions and regulatory mechanisms of intratumoural fungi in EOC remain poorly characterised. In this study, we systematically profiled the intratumoural fungal community in EOC and explored its potential roles and underlying molecular mechanisms in driving tumour progression.

To characterise the intratumoural mycobiota in patients with EOC and EBOT, we first confirmed the presence of fungal signals in tumour tissues by 28S rRNA FISH, with more prominent signals detected in EOC tissues than in EBOT tissues. We then performed comparative analyses of fungal diversity and composition using ITS1 rDNA sequencing. Our results revealed profound dysbiosis within the EOC niche, characterised by a significant reduction in α‐diversity and distinct β‐diversity clustering relative to EBOT. At the genus level, the most striking feature was the marked enrichment of *Malassezia* in EOC tissues (Figure 1, Figure S1). Such fungal dysbiosis is increasingly recognised as a hallmark of multiple malignancies (Sun et al. 2025). Recent large‐scale mycobiome atlases have identified tumour‐resident fungal communities in pancreatic (Aykut et al. 2019), colorectal (Yuan et al. 2025) and lung cancers (Liu, Li, et al. 2023; Liu, Yi, et al. 2023), positioning these fungi as active contributors to disease pathogenesis. Notably, *Malassezia* has emerged as a particularly prominent genus implicated in diverse oncogenic processes. For example, Zhang et al. reported that *Malassezia* enrichment in hepatocellular carcinoma correlates with poor prognosis by downregulating bile acid synthesis enzymes, thereby reshaping the metabolic landscape of the TME (Shen et al. 2025). Similar associations have been described in colorectal and oral cancers, where *Malassezia* abundance correlates with increased pro‐inflammatory cytokine secretion and unfavourable clinical outcomes (Mohamed et al. 2021; Liu, Li, et al. 2023; Liu, Yi, et al. 2023; Yuan et al. 2025). Consistent with these observations, *Malassezia* enrichment in EOC tissues in our study was positively correlated with serum CA125 levels and malignant progression, suggesting that this genus may exert tumour‐promoting effects across distinct cancer types. Collectively, these findings indicate that *Malassezia* may play an active oncogenic role in EOC progression.

To further elucidate the contribution of intratumoural microbiota to EOC progression, we established a murine EOC model in which endogenous microbiota derived from patients with EBOT or EOC were administered intratumourally. Consistent with our clinical observations, both microbiota mixtures significantly promoted tumour growth (Figure 2). Notably, EOC‐derived microbiota exerted a more pronounced tumour‐promoting effect than EBOT‐derived microbiota, suggesting that tumour‐specific microbial communities may possess distinct functional properties that uniquely modulate the TME. Of particular interest, *M. restricta* alone induced tumour‐promoting effects comparable to those observed with EOC‐derived microbiota (Figure 3). These findings align with previous reports demonstrating that *M. restricta* accelerates tumour growth by modulating immune responses and promoting an inflammatory TME (Gamal et al. 2022). In pancreatic cancer, *M. restricta* has been shown to promote tumour progression by enhancing immune suppression through interactions with the host immune system (Aykut et al. 2019; Brayer et al. 2023). A similar mechanism may operate in EOC, whereby *M. restricta* reshapes local immune responses to create a permissive environment for tumour growth.

The TME is a critical regulator of tumour progression, with immune cells playing central roles in shaping disease outcomes. Increasing evidence suggests that intratumoural fungi influence immune cell composition and function, thereby promoting cancer progression (Li and Saxena 2022; Ding et al. 2025; Mou et al. 2025). Among immune cell populations, TAMs are key components of the TME and exhibit substantial phenotypic plasticity (Chen et al. 2021). These cells are broadly classified into two functional states: M1 macrophages, which secrete pro‐inflammatory mediators such as TNF‐α and IL‐6 and exert anti‐tumour activity (Wang, Wang, et al. 2023; Wang, Wu, et al. 2023), and M2 macrophages, which constitute the predominant macrophage population in most solid tumours and contribute to an immunosuppressive microenvironment (Hong et al. 2024; Xu, Ding, et al. 2025; Xu, Luo, et al. 2025; Xu, Qiao, et al. 2025). M2 macrophages are characterised by the production of anti‐inflammatory cytokines, including TGF‐β and IL‐10, and by expression of markers such as CD206 and Arg1 (Basak et al. 2023; Chen et al. 2024). These cells promote tumour progression by suppressing anti‐tumour immunity and facilitating angiogenesis and tissue remodelling (Han et al. 2023). Wang, Wang, et al. (2023) and Wang, Wu, et al. (2023) showed that *C. albicans* drives M2 polarisation of TAMs via the IL‐17A/PD‐L1 axis, thereby promoting oral cancer progression. Similarly, Liu et al. (2024) reported that *M. globosa* accumulation in breast cancer tissues induces M2 macrophage polarisation through IL‐17A signalling, accelerating tumour growth. In line with these reports, our data demonstrate that *M. restricta* colonisation in EOC tissues significantly increased the proportion of tumour‐infiltrating M2 macrophages, while the levels of M1 macrophages, CD4^+^ T cells and CD8^+^ T cells remained largely unchanged. These findings further underscore the importance of M2 macrophage polarisation in EOC progression. Moreover, *M. restricta* colonisation led to increased expression of CD206 and Arg1, accompanied by elevated levels of TGF‐β and IL‐10 in murine tumours (Figure 3, Figure S2), consistent with the established functional profile of M2 macrophages. Nevertheless, the effects of *M. restricta* on other immune cell subsets, including dendritic cells, natural killer cells and B cells, remain to be determined and warrant further investigation.

To identify the *M. restricta* components responsible for inducing M2 macrophage polarisation, the culture supernatant and heat‐inactivated cell pellets of *M. restricta* were isolated by centrifugation and co‐cultured with RAW264.7 cells. Notably, exposure to the culture supernatant markedly enhanced M2 macrophage differentiation compared with the control condition, whereas the heat‐inactivated cell pellets had no such effect. These findings indicate that the *M. restricta* culture supernatant contains key effector molecules capable of promoting M2 macrophage polarisation (Figure 4). EVs are bilayer membrane nanostructures released by microorganisms during growth and contain a diverse range of bioactive components, including nucleic acids, proteins, enzymes and lipids (van Niel et al. 2018; Wang, Wang, et al. 2025; Wang, Zhu, et al. 2025). Increasing evidence indicates that microbial EVs modulate immune cell function and contribute to tumour progression (Zhang and Yu 2019; Kuang et al. 2025). For example, Choi et al. (2017) demonstrated that EVs derived from *Helicobacter pylori* carry the virulence factors CagA and VacA, activate the NF‐κB pathway in gastric epithelial cells, and induce pro‐inflammatory cytokines such as IL‐8 and TNF‐α, thereby promoting gastric cancer progression. In the present study, EVs were successfully isolated from the culture supernatant of *M. restricta*. TEM analysis confirmed that purified MrEVs displayed a typical bilayer membrane structure with round or elliptical morphology. Furthermore, our data showed that macrophages readily internalised MrEVs, which in turn promoted M2 polarisation and increased production of TGF‐β and IL‐10 in vitro (Figure 5, Figure S3). Together, these findings identify MrEVs as critical intermediaries in *M. restricta*‐mediated M2 macrophage polarisation and underscore the importance of further investigating their biological functions and underlying mechanisms.

To elucidate the molecular pathways through which MrEVs drive M2 macrophage differentiation, RT‐qPCR analysis was performed to assess the expression of key genes associated with macrophage polarisation. Among the candidates examined, only TLR4 and JAK2 showed significant upregulation following MrEV treatment compared with the control group (Figure S4). The selective induction of TLR4 suggests that MrEVs may contain fungal‐derived ligands, such as specific glycoproteins or lipids, capable of engaging this receptor (Janssens and Beyaert 2003; Su et al. 2021; Honorato et al. 2024). Meanwhile, increased JAK2 expression is consistent with previous studies highlighting its central role in M2 macrophage polarisation (Kerneur et al. 2022). As a major downstream effector of JAK2, STAT3 is widely recognised as a key transcriptional regulator of the M2 phenotype (Irey et al. 2019; Xia et al. 2023). The JAK2/STAT3 axis has been proposed to function as a molecular switch directing macrophage polarisation towards the M2 state (Zeng et al. 2022; Li et al. 2023). Supporting this concept, Xiao et al. (2024) showed that gastric cancer cell‐derived exosomes deliver miR‐541‐5p to macrophages, activating the JAK2/STAT3 pathway and promoting M2 polarisation. Similarly, Yang et al. demonstrated that EVs released from hepatic stellate cells induce M2 polarisation of Kupffer cells via JAK2/STAT3 signalling, with phosphorylated STAT3 translocating to the nucleus and binding to the IL‐10 promoter to drive transcription and fibrotic progression (Yang et al. 2024). Based on these observations, we hypothesised that MrEVs promote M2 macrophage polarisation through activation of the JAK2/STAT3 signalling pathway. Consistent with this hypothesis, MrEV treatment markedly increased the expression levels of Arg1, JAK2, and STAT3 compared with controls. Collectively, these findings indicate that MrEVs induce M2 macrophage polarisation through specific activation of the JAK2/STAT3 pathway. Despite these insights, the precise molecular components within MrEVs responsible for triggering JAK2/STAT3 activation remain unidentified. In particular, whether MrEVs contain miRNAs that directly target components of the JAK2/STAT3 pathway warrants further investigation using approaches such as small RNA sequencing and dual‐luciferase reporter assays. Identification of these active molecules would not only clarify the effector–pathway axis underlying MrEV function, but also provide potential targets for disrupting fungal EV‐mediated immune evasion.

To further validate the role of the JAK2/STAT3 pathway in M2 macrophage polarisation, AG490 was employed as a JAK2 inhibitor in both cellular and animal models. AG490 is a widely used and selective inhibitor of JAK2 and has been extensively applied to interrogate JAK2/STAT3‐dependent biological processes (Kobayashi et al. 2015; Fang et al. 2024). Previous studies have shown that IL‐17A promotes colon cancer malignancy through activation of the JAK2/STAT3 pathway, an effect that can be counteracted by AG490 (Zhang et al. 2020). In addition, Yang et al. reported that activated hepatic stellate cells induce liver fibrosis via JAK2/STAT3‐dependent M2 macrophage polarisation and that AG490 suppresses this process by targeting JAK2/STAT3 signalling (Yang et al. 2024). In line with these reports, our results demonstrated that AG490 effectively inhibited JAK2 activation. Both in vitro and in vivo experiments showed that AG490 suppressed JAK2/STAT3 signalling, reduced M2 macrophage polarisation, and ultimately delayed tumour progression (Figures 6 and 7). These findings further support the conclusion that *M. restricta* promotes tumour progression by driving M2 macrophage polarisation through the JAK2/STAT3 signalling pathway.

Several limitations of this study should be acknowledged. First, the relatively small number of EBOT and EOC samples necessitates validation in larger patient cohorts. In addition, analysis of *Malassezia* abundance across different pathological stages would help confirm its enrichment during EOC progression. Second, the colonisation burden of *Malassezia* in patients and mouse models was not quantitatively assessed; future studies employing digital PCR or metagenomic approaches would allow more precise quantification. Third, germ‐free animal models should be used in future experiments to eliminate potential confounding effects of endogenous fungi. Fourth, although MrEVs were identified as key immunomodulatory effectors, the specific molecular components responsible for TLR4 activation remain unknown. Integrating component depletion strategies with proteomic and RNA sequencing approaches may help identify these active molecules. Finally, because AG490 did not completely suppress JAK2 activity, genetic approaches such as JAK2 knockout models will be required to further validate the role of this pathway.

## 5 Conclusion

**FIGURE 8:**
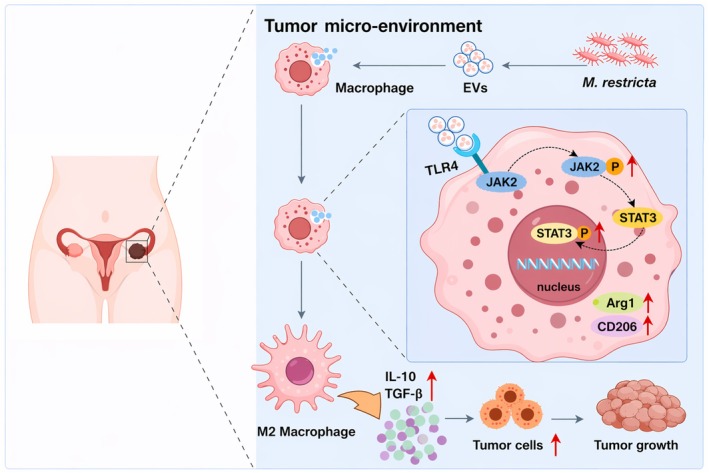
Schematic diagram of the potential mechanism by which *Malassezia restricta* promotes ovarian cancer progression.

In summary, this study demonstrates for the first time that intratumoural *M. restricta* significantly accelerates EOC progression and enhances M2 macrophage infiltration within the TME. Furthermore, EVs derived from *M. restricta* promote tumour progression by driving M2 macrophage polarisation through activation of the JAK2/STAT3 signalling pathway (Figure 8). Together, these findings elucidate a previously unrecognised link between intratumoural fungi and EOC progression, advancing our understanding of the fungal EV–immune axis in EOC pathogenesis. This work provides a foundation for future investigations into the role of intratumoural mycobiota in cancer progression and highlights potential targets for therapeutic intervention.

## Author Contributions

**Ying Jiang:** conceptualization, writing – original draft, visualization, investigation. **Fen Wei:** methodology. **Qingling Yang:** software. **Xue Wu:** data curation. **Qifa Huang:** investigation. **Ang Dai:** supervision. **Qi Chen:** funding acquisition, conceptualization. **Tingtao Chen:** conceptualization, writing – review and editing.

## Funding

This work was supported by the National Natural Science Foundation of China (No. 82260507).

## Ethics Statement

Ethical oversight for the human portion of this research was provided by the Second Affiliated Hospital of Nanchang University's medical research ethics committee (No. Review [2024] 076), with all procedures aligning with the Declaration of Helsinki. Regarding animal welfare, the study protocol was authorised by the Animal Ethics Committee of Nanchang University under the reference NCULAE‐20221031067.

## Conflicts of Interest

The authors declare no conflicts of interest.

## Supporting information


**Figure S1:** Intratumoural mycobiome dysbiosis in patients with EOC. (A) Fluorescence in situ hybridization (FISH) illustrating presence of fungi in EBOT tissue and EOC tissue. Scale bar: 50 μm. (B) Relative abundance of *Malassezia* in EBOT and EOC tissues determined by qPCR. Data are shown as mean ± SD. **p < 0.01.
**Figure S2:** *Malassezia restricta* colonisation promotes EOC progression and enhances M2 macrophage infiltration in the TME. (A) IF staining for CD4 and CD8. Scale bar: 50 μm. (B–G) mRNA levels of *CD86*, *iNOS*, *IL‐17A*, *FOXP3*, *IFNG* and *GZMB* (*n* = 4). (H, I) Levels of IL‐6, TNF‐α (*n* = 4). Groups: M (ID8 tumour‐bearing model group); MT (*M. restricta* colonisation); ET (EOC‐derived microbial transfer). Data are shown as mean ± SD. *p < 0.05.
**Figure S3:** EVs from *M. restricta* are internalised by macrophages and promote M2 polarisation. (A) Flow chart of the cell experiment design. (B) IF staining for CD206 in RAW 264.7 cells. Scale bar: 10 μm. (C, D) mRNA levels of *CD206* and *Arg1* (*n* = 4). (E, F) Protein levels of Arg1 (*n* = 4). (G, H) Levels of IL‐10, TGF‐β (*n* = 4). Groups: C (control group); S (treated with *M. restricta* culture supernatant for 24 h); EVs (treated with MrEVs at 20 μg/mL for 24 h). Data are shown as mean ± SD. **p < 0.01, ***p < 0.001.
**Figure S4:** EVs from *M. restricta* are internalised by macrophages and promote M2 polarisation. (A–H) mRNA levels of *TLR2*, *TLR4*, *Dectin‐1*, *AKT*, *JAK2, ERK, p38MAPK* and *β‐catenin* (*n* = 4). Groups: C (control group); EVs (treated with MrEVs at 20 μg/mL for 24 h). Data are shown as mean ± SD. ns, not significant, **p < 0.01, ***p < 0.001.

**Table S1:** Cell line metadata and authentication status.

**Table S2:** Chemicals information.

**Table S3:** Primer sequences of RT‐qPCR.

**Table S4:** Antibodies used in the article.

**Table S5:** Baseline characteristics of patients with EBOT and EOC.

## Data Availability

The ITS1 rDNA sequencing data generated in this study have been deposited in the NCBI Sequence Read Archive (SRA) under BioProject accession number PRJNA1369007 (https://www.ncbi.nlm.nih.gov/). All other data supporting the findings of this study are included in the Supporting Information or are available from the corresponding author upon reasonable request.
