# Supplementary material for: Malassezia restricta‐Derived Extracellular Vesicles Drive Ovarian Cancer Progression Through JAK2/STAT3‐Mediated M2 Macrophage Polarisation
Source: Microb Biotechnol. 2026 Jun 5;19(6):e70396. doi: 10.1111/1751-7915.70396 (PMC13241584; doi:10.1111/1751-7915.70396)
Supplement: Supplementary file 1 — Figure S1: Intratumoural mycobiome dysbiosis in patients with EOC. (A) Fluorescence in situ hybridization (FISH) illustrating presence of fungi in EBOT tissue and EOC tissue. Scale bar: 50 μm. (B) Relative abundance of Malassezia in EBOT and EOC tissues determined by qPCR. Data are shown as mean ± SD. **p < 0.01. Figure S2: Malassezia restricta colonisation promotes EOC progression and enhances M2 macrophage infiltration in the TME. (A) IF staining for CD4 and CD8. Scale bar: 50 μm. (B–G) mRNA levels of CD86, iNOS, IL‐17A, FOXP3, IFNG and GZMB (n = 4). (H, I) Levels of IL‐6, TNF‐α (n = 4). Groups: M (ID8 tumour‐bearing model group); MT (M. restricta colonisation); ET (EOC‐derived microbial transfer). Data are shown as mean ± SD. *p < 0.05. Figure S3: EVs from M. restricta are internalised by macrophages and promote M2 polarisation. (A) Flow chart of the cell experiment design. (B) IF staining for CD206 in RAW 264.7 cells. Scale bar: 10 μm. (C, D) mRNA levels of CD206 and Arg1 (n = 4). (E, F) Protein levels of Arg1 (n = 4). (G, H) Levels of IL‐10, TGF‐β (n = 4). Groups: C (control group); S (treated with M. restricta culture supernatant for 24 h); EVs (treated with MrEVs at 20 μg/mL for 24 h). Data are shown as mean ± SD. **p < 0.01, ***p < 0.001. Figure S4: EVs from M. restricta are internalised by macrophages and promote M2 polarisation. (A–H) mRNA levels of TLR2, TLR4, Dectin‐1, AKT, JAK2, ERK, p38MAPK and β‐catenin (n = 4). Groups: C (control group); EVs (treated with MrEVs at 20 μg/mL for 24 h). Data are shown as mean ± SD. ns, not significant, **p < 0.01, ***p < 0.001. [file MBT2-19-e70396-s002.docx]

### **Supplementary material**


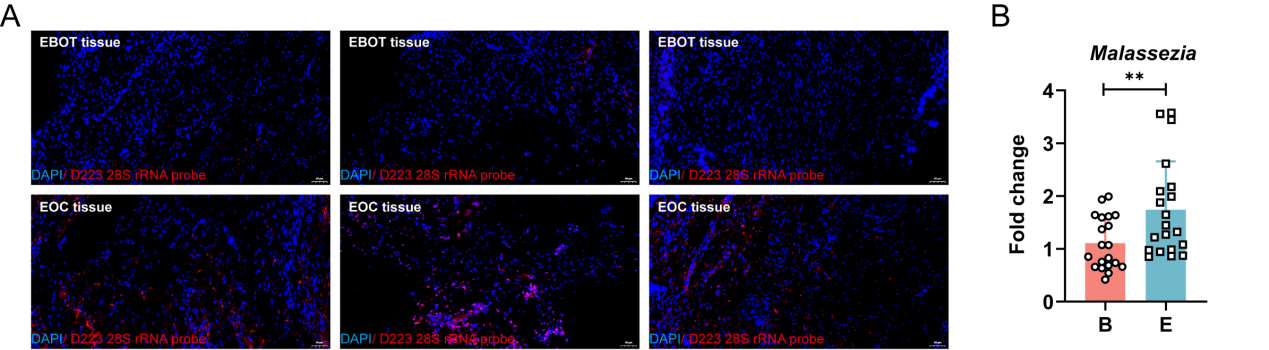


**Supplementary Figure 1. Intratumoural Mycobiome Dysbiosis in Patients with EOC. (A)** Fluorescence in situ hybridization (FISH) illustrating presence of fungi in EBOT tissue and EOC tissue. Scale bar: 50 μm. **(B)** Relative abundance of *Malassezia* in EBOT and EOC tissues determined by qPCR. Data are shown as mean ± SD. * *p* < 0.05, ** *p* < 0.01, *** *p* < 0.001.

**
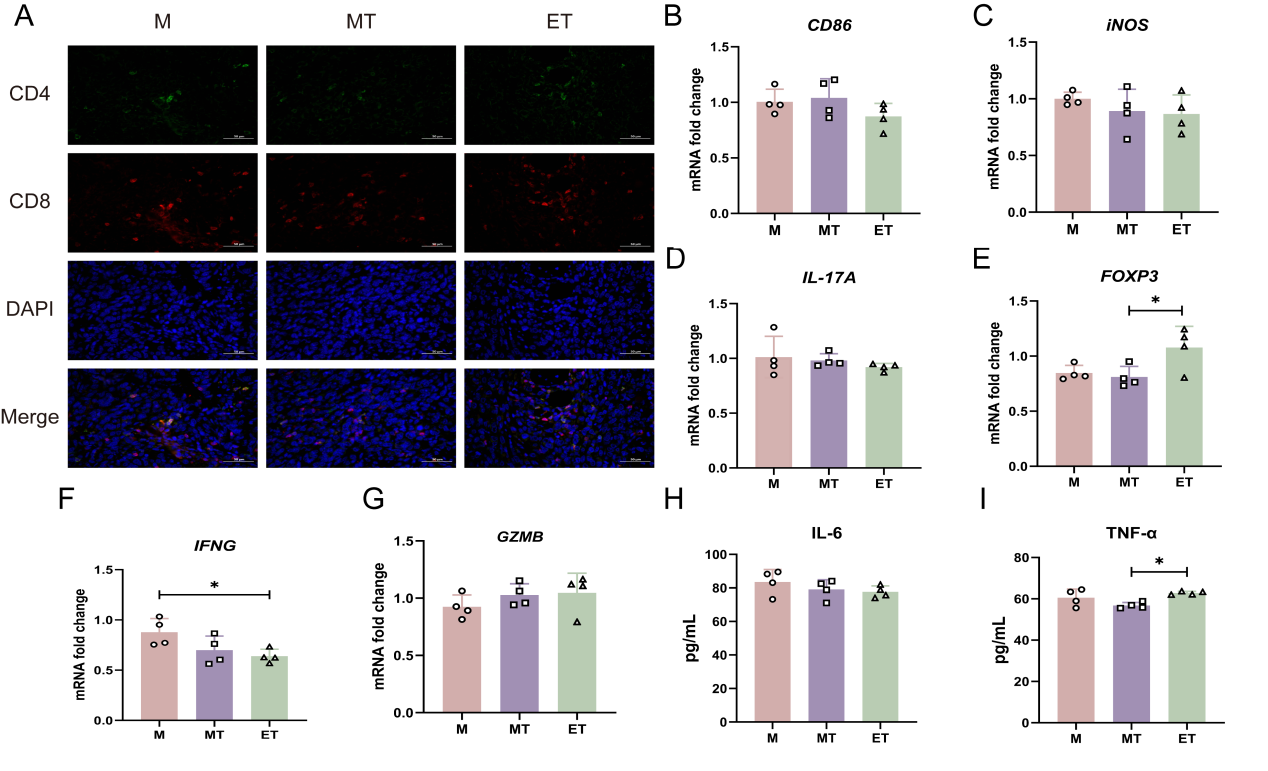
**

**Supplementary Figure 2. *Malassezia restricta* Colonisation Promotes EOC Progression and Enhances M2 Macrophage Infiltration in the TME. (A)** IF staining for CD4 and CD8. Scale bar: 50 μm. **(B-G)** mRNA levels of *CD86*, *iNOS*, *IL-17A*, *FOXP3*, *IFNG*, and *GZMB* (n = 4). **(H-I)** levels of IL-6, TNF-α (n = 4). Groups: M (ID8 tumour-bearing model group); MT (M. restricta colonization); ET (EOC-derived microbial transfer). Data are shown as mean ± SD. * *p* < 0.05, ** *p* < 0.01, *** *p* < 0.001.


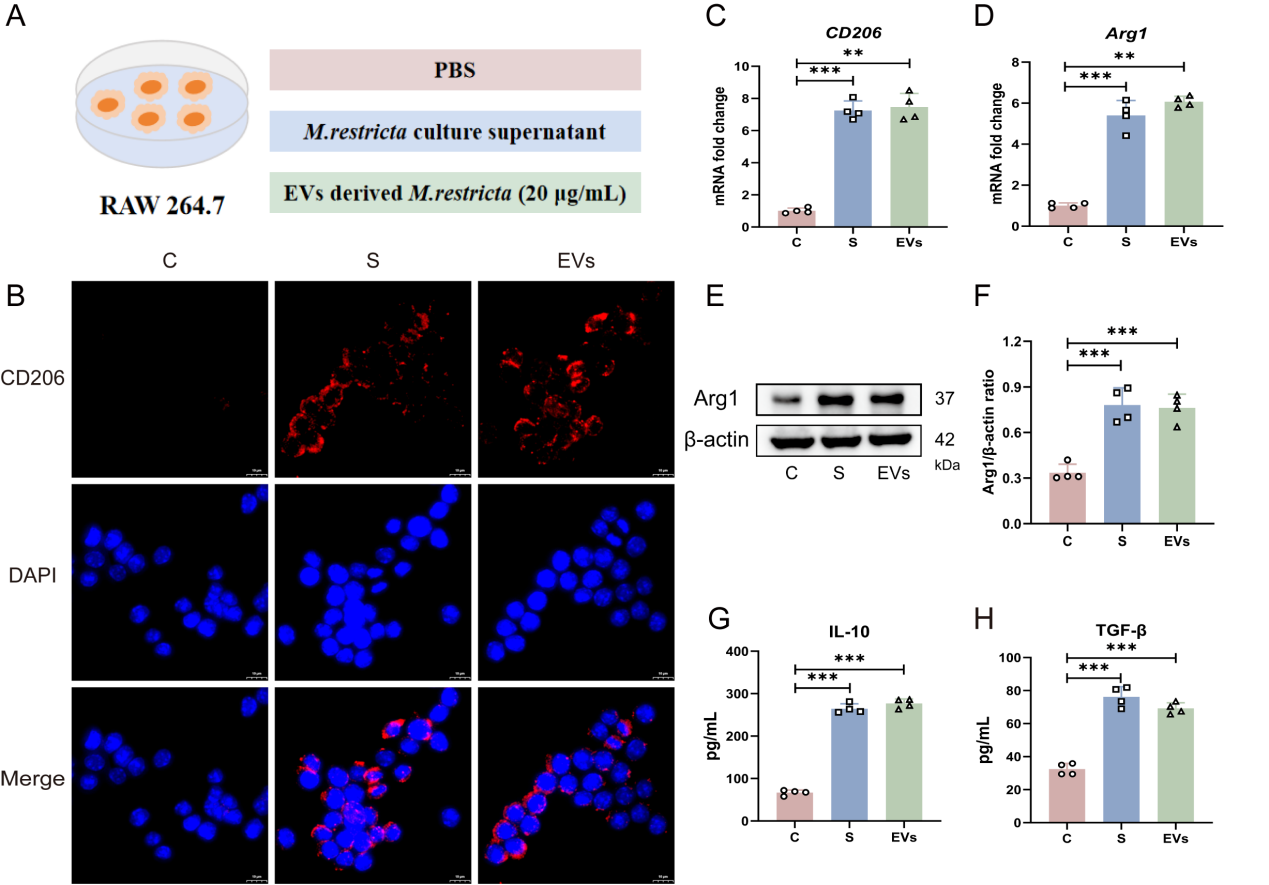


**Supplementary Figure 3. EVs from M. restricta are Internalised by Macrophages and Promote M2 Polarisation. (A)** Flowchart of the cell experiment design. **(B)** IF staining for CD206 in RAW 264.7 cells. Scale bar: 10 μm. **(C-D)** mRNA levels of *CD206* and *Arg1* (n = 4). **(E-F)** Protein levels of Arg1 (n = 4). **(G-H)** levels of IL-10, TGF-β (n = 4). Groups: C (control group); S (treated with *M. restricta* culture supernatant for 24 h); EVs (treated with MrEVs at 20 μg/mL for 24 h). Data are shown as mean ± SD. * *p* < 0.05, ** *p* < 0.01, *** *p* < 0.001.


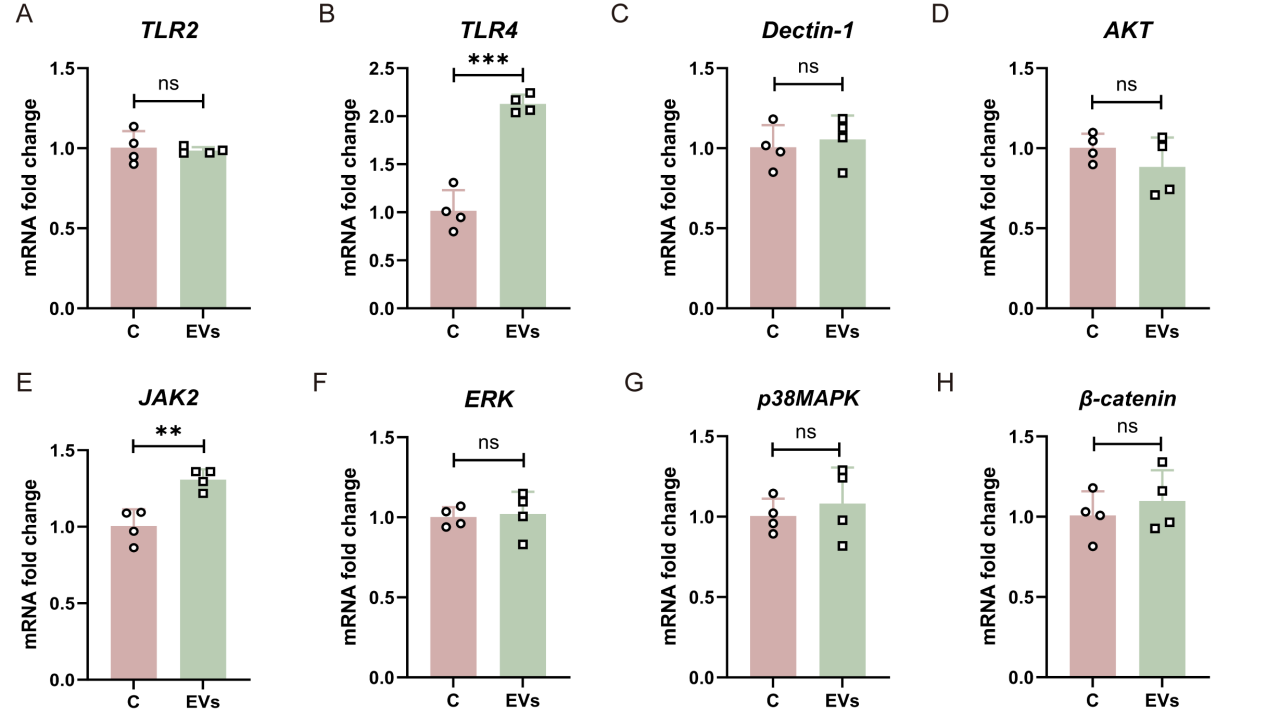


**Supplementary Figure 4. EVs from M. restricta Are Internalised by Macrophages and Promote M2 Polarisation. (A-H)** mRNA levels of *TLR2*, *TLR4*, *Dectin-1*, *AKT*, *JAK2, ERK, p38MAPK* and *β-catenin* (n = 4). Groups: C (control group); EVs (treated with MrEVs at 20 μg/mL for 24 h). Data are shown as mean ± SD. * *p* < 0.05, ** *p* < 0.01, *** *p* < 0.001.
