# Supplementary material for: Malassezia restricta‐Derived Extracellular Vesicles Drive Ovarian Cancer Progression Through JAK2/STAT3‐Mediated M2 Macrophage Polarisation
Source: Microb Biotechnol. 2026 Jun 5;19(6):e70396. doi: 10.1111/1751-7915.70396 (PMC13241584; doi:10.1111/1751-7915.70396)
Supplement: Supplementary file 2 — Table S1: Cell line metadata and authentication status. [file MBT2-19-e70396-s004.docx]

**Supplementary Table 1. Cell Line Metadata and Authentication Status**

| **Feature** | **ID8** | **RAW 264.7** |
| --- | --- | --- |
| **Official Name** | ID8 | RAW 264.7 |
| **RRID** | CVCL_IU14 | CVCL_0493 |
| **Species** | *Mus musculus* (Mouse) | *Mus musculus* (Mouse) |
| **Sex** | Female | Male |
| **Tissue of Origin** | Ovarian surface epithelium | Blood / Ascites |
| **Supplier** | Shanghai Fuxiang Biotechnology | Wuhan Servicebio Technology |
| **Catalog Number** | XF1030 | STCC20020 |
| **Obtained Date** | May 2019 | August 2024 |
| **Authentication Method** | STR Profiling(20 loci) | STR Profiling (18 loci) |
| **% Match Result** | 100% | 95.20% |
| **Mycoplasma Status** | Negative | Negative |
| **Problematic Line (ICLAC)** | No | No |
