# Supplementary material for: Malassezia restricta‐Derived Extracellular Vesicles Drive Ovarian Cancer Progression Through JAK2/STAT3‐Mediated M2 Macrophage Polarisation
Source: Microb Biotechnol. 2026 Jun 5;19(6):e70396. doi: 10.1111/1751-7915.70396 (PMC13241584; doi:10.1111/1751-7915.70396)
Supplement: Supplementary file 3 — Table S2: Chemicals information. [file MBT2-19-e70396-s003.docx]

**Supplementary Table 2. Chemicals information**

| Chemicals |  |  |
| --- | --- | --- |
| IL6 ELISA Kit | MultiSciences | Cat# EK206 |
| TNF-α ELISA Kit | MultiSciences | Cat# EK282 |
| IL10 ELISA Kit | MultiSciences | Cat# EK210 |
| TGF-β ELISA Kit | MultiSciences | Cat# EK981 |
| PrimeScript™RT Reagent Kit with gDNA Eraser | Takara | Cat# RR047A |
| TB Green®Premix Ex Taq™ II | Takara | Cat# RR820A |
| Bicinchoninic Acid Kit | Beyotime | Cat#P0010 |
| CCK-8 kit | Beyotime | Cat#C0038 |
| Tyrphostin AG490 | MCE | Cat#HY-12000 |
