# Supplementary material for: Malassezia restricta‐Derived Extracellular Vesicles Drive Ovarian Cancer Progression Through JAK2/STAT3‐Mediated M2 Macrophage Polarisation
Source: Microb Biotechnol. 2026 Jun 5;19(6):e70396. doi: 10.1111/1751-7915.70396 (PMC13241584; doi:10.1111/1751-7915.70396)
Supplement: Supplementary file 4 — Table S3: Primer sequences of RT‐qPCR. [file MBT2-19-e70396-s005.docx]

**Supplementary Table 3 Primer sequences of RT-qPCR**

| Species | Gene | Description | Primer (5′-3′) |
| --- | --- | --- | --- |
| Mouse | *CD206* | Forward  Reverse | GAGGGAAGCGAGAGATTATGGA  GCCTGATGCCAGGTTAAAGCA |
|  | *Arg1* | Forward  Reverse | AGACAGCAGGGAGGTGAAGAG  CGAAGCAAGCAAGGTTAAAGC |
|  | *CD86* | Forward  Reverse | TTGTGTGTGTTCTGGAAACGGAG  AACTTAGAGGCTGTGTTGCTGGG |
|  | *iNOS* | Forward  Reverse | GGGAATCTTGGAGCGAGTTGT  GCACATGCAAGGAAGGGAAC |
|  | *IFNG* | Forward  Reverse | ATGAACGCTACACACTGCATC  CCATCCTTTTGCCAGTTCCTC |
|  | *GZMB* | Forward  Reverse | AGAAAGACAGTCACAGGCCAAA  GTCTTCTTCCGTGCTTCATCCA |
|  | *IL-17A* | Forward  Reverse | TCCAGAAGGCCCTCAGACTA  AGCATCTTCTCGACCCTGAA |
|  | *FOXP3* | Forward  Reverse | CCCAGGAAAGACAGCAACCTT  TTGTCACCTAGGCCACTTGAG |
|  | *TLR2* | Forward  Reverse | GCAAACGCTGTTCTGCTCAG  AGGCGTCTCCCTCTATTGTATT |
|  | *TLR4* | Forward  Reverse | ATGGCATGGCTTACACCACC  GAGGCCAATTTTGTCTCCACA |
|  | *Dectin-1* | Forward  Reverse | TTCTCAGCCTTGCCTTCCTA  TACGGTGAGACGATGTTTGG |
|  | *AKT* | Forward  Reverse | GAAGTTGCTCTACCCAGTGTCC  GATAGCCGTTCCTTTCATTTGG |
|  | *JAK2* | Forward  Reverse | TGGAGCTCAAATCTCGAACGA  TGAATTTCCTGCTCCTGAAGC |
|  | *ERK* | Forward  Reverse | AGGTTGTTCCCAAACGCTGA  AGGTAAGTCGTCCAGCTCCA |
|  | *p38MAPK* | Forward  Reverse | GCATCATGGCTGAGCTGTTG  GAGATAAGCAGGGGGTGTCC |
|  | *β-catenin* | Forward  Reverse | TGCAGTGGGTACTCAGGAAAG  GATTCTGAGAGCGGCAAGA |
|  | *β-actin* | Forward  Reverse | CTACCTCATGAAGATCCTGACC  CACAGCTTCTCTTTGATGTCAC |
|  | 18S rDNA | Forward  Reverse | CGGTAACCCGCTGAACTTAA  GGCATCACAGACCTGTTATT |
|  | 1. *restricta* | Forward  Reverse | GGAAGTAAAAGTCGTAACAAGG  CCTCCGCTTATTGATATGC |
