# Supplementary material for: Malassezia restricta‐Derived Extracellular Vesicles Drive Ovarian Cancer Progression Through JAK2/STAT3‐Mediated M2 Macrophage Polarisation
Source: Microb Biotechnol. 2026 Jun 5;19(6):e70396. doi: 10.1111/1751-7915.70396 (PMC13241584; doi:10.1111/1751-7915.70396)
Supplement: Supplementary file 5 — Table S4: Antibodies used in the article. [file MBT2-19-e70396-s001.docx]

**Supplementary Table 4. Antibodies used in the article**

| Target antigen | Vendor | Catalog# | Concentration |
| --- | --- | --- | --- |
| anti-β-Actin antibody | ProteinTech | 20536-1-AP | 1:5000 |
| anti-iNOS antibody | ProteinTech | 18985-1-AP | 1:1000 |
| anti-Arg1 antibody | ProteinTech | 16001-1-AP | 1:5000 |
| anti-CD86 antibody | ABclonal | A16805 | 1:100 |
| anti-CD206 antibody | ABclonal | A21014 | 1:100 |
| anti-F4/80 antibody | ProteinTech | 28463-1-AP | 1:100 |
| anti-CD4 antibody | ABclonal | A0362 | 1:100 |
| anti-CD8a antibody | ProteinTech | 29896-1-AP | 1:100 |
| anti-TLR4 antibody | ABclonal | A11226 | 1:500 |
| anti-JAK2 antibody | UpingBio | YP-mAb-14800 | 1:1000 |
| anti-p-JAK2 antibody | UpingBio | YP-mAb-14359 | 1:1000 |
| anti-STAT3 antibody | ABclonal | A1192 | 1:1000 |
| anti-p-STAT3 antibody | ABclonal | AP0474 | 1:1000 |
