# Supplementary material for: Malassezia restricta‐Derived Extracellular Vesicles Drive Ovarian Cancer Progression Through JAK2/STAT3‐Mediated M2 Macrophage Polarisation
Source: Microb Biotechnol. 2026 Jun 5;19(6):e70396. doi: 10.1111/1751-7915.70396 (PMC13241584; doi:10.1111/1751-7915.70396)
Supplement: Supplementary file 6 — Table S5: Baseline characteristics of patients with EBOT and EOC. [file MBT2-19-e70396-s006.docx]

| **Supplementary Table 5. Baseline characteristics of patients with EBOT and EOC.** | | | |
| --- | --- | --- | --- |
|  | **EBOT(n=20)** | **EOC(n=20)** | ***p*-value** |
| Age(years) | 53.65±11.03 | 60.00±9.570 | *P=0.059* |
| BMI (kg/m^2^) | 23.26±3.832 | 22.12±2.556 | *P=0.147* |
| Current smoker |  |  |  |
| No | 18(90%) | 19(95%) | *p=0.500* |
| Yes | 2(10%) | 1(5%) |  |
| Age at menarche (years) |  |  | *p=0.723* |
| ≤12 | 5(25%) | 6(30%) |  |
| >12 | 15(75%) | 14(70%) |  |
| Menopausal status |  |  | *p=0.705* |
| Premenopausal | 5(25%) | 4(20%) |  |
| Postmenopausal | 15(75%) | 16(80%) |  |
| Current OCP use |  |  | *p=0.500* |
| No | 18(90%) | 19(95%) |  |
| Yes | 2(10%) | 1(5%) |  |
| Ever pregnant |  |  | *p=0.756* |
| No | 1(5%) | 1(5%) |  |
| Yes | 19(95%) | 19(95%) |  |
| Tubal ligation |  |  | *p=0.337* |
| No | 13(65%) | 10(50%) |  |
| Yes | 7(35%) | 10(50%) |  |
| Serum CA125  Median (P25, P75) | 14.80 (10.05, 21.40) | 308.7 (74.83, 1792) | *p＜0.001* |

EOC = epithelial ovarian cancer; EBOT = epithelial benign ovarian tumor; BMI = body mass index; OCP = oral contraceptive pill. Data are expressed as n (%), mean ± standard deviation, or median (interquartile range). Statistical significance was set at two-tailed *p* < 0.05. Group comparisons were made using independent samples t-test for normally distributed variables, Mann-Whitney U test for non-normally distributed variables, and Chi-square (χ²) test for categorical variables
